# Genome-wide meta-analysis of 92 cardiometabolic protein serum levels

**DOI:** 10.1016/j.molmet.2023.101810

**Published:** 2023-09-29

**Authors:** Arthur Gilly, Young-Chan Park, Emmanouil Tsafantakis, Maria Karaleftheri, George Dedoussis, Eleftheria Zeggini

**Affiliations:** 1 Institute of Translational Genomics, Helmholtz Zentrum München – German Research Center for Environmental Health, Neuherberg, Germany; 2 Anogia Medical Centre, Anogia, Greece; 3 Echinos Medical Centre, Echinos, Greece; 4 Department of Nutrition and Dietetics, School of Health Science and Education, Harokopio University of Athens, Athens, Greece; 5 Technical University of Munich (TUM) and Klinikum Rechts der Isar, TUM School of Medicine, Munich, Germany

**Keywords:** Proteomics, Cardiometabolic diseases, Genome-wide association study, Quantitative trait loci, Isolated populations

## Abstract

**Objectives:**

Global cardiometabolic disease prevalence has grown rapidly over the years, making it the leading cause of death worldwide. Proteins are crucial components in biological pathways dysregulated in disease states. Identifying genetic components that influence circulating protein levels may lead to the discovery of biomarkers for early stages of disease or offer opportunities as therapeutic targets.

**Methods:**

Here, we carry out a genome-wide association study (GWAS) utilising whole genome sequencing data in 3,005 individuals from the HELIC founder populations cohort, across 92 proteins of cardiometabolic relevance.

**Results:**

We report 322 protein quantitative trait loci (pQTL) signals across 92 proteins, of which 76 are located in or near the coding gene (*cis*-pQTL). We link those association signals with changes in protein expression and cardiometabolic disease risk using colocalisation and Mendelian randomisation (MR) analyses.

**Conclusions:**

The majority of previously unknown signals we describe point to proteins or protein interactions involved in inflammation and immune response, providing genetic evidence for the contributing role of inflammation in cardiometabolic disease processes.

## Highlights

• Whole-genome sequencing gives access to insights into new protein biology.
• *Trans-*pQTL resolved using eQTL confirm experimental interactions (e.g. CCL5/ACKR1).
• Genetic associations linking cardiometabolic proteins reflect inflammation pathways.

## List of abbreviations

2SMR: two-sample Mendelian randomisation
CCL5: C–C Motif Chemokine Ligand 5
CMET: Cardiometabolic
COJO: conditional and joint association analysis
DARC: Duffy antigen receptor for chemokines
EAF: effect allele frequency
eQTL: expression quantitative trait loci
GAS6: Growth Arrest Specific 6
GATK: Genome Analysis Toolkit
GP: glycoprotein
GP1BA: glycoprotein Ib alpha chain
GWAS: genome-wide association study
HELIC: Hellenic Isolated Cohort
Ig: Immunoglobulin
IGLC2: Immunoglobulin lambda light chain constant 2
INDEL: insertion or deletion
LD: linkage disequilibrium
LOD: lower limit of detection
MAF: minor allele frequency
MFAP5: Microfibrillar-associated protein 5
MR: Mendelian randomisation
NPX: Normalised Protein Expression
pQTL: protein quantitative trait loci
SNVs: single nucleotide variants
T2D: type 2 diabetes
TAA: thoracic aortic aneurysm
TIMP3: tissue inhibitor of matrix metalloproteinases
UKBB: UK Biobank
VQSR: Variant Quality Score Recalibration
VWF: von Willebrand factor
WGS: whole genome sequence

## 1 Introduction

Cardiometabolic diseases are common, complex conditions that include cardiovascular disease, diabetes mellitus, insulin resistance and non-alcoholic fatty liver disease. These disorders, which impose a growing health burden on modern societies, often have a chronic inflammation component. Pre-existing chronic inflammation is thought to accelerate the development of these diseases, and targeting systemic inflammation in humans is increasingly seen as a promising approach for prevention and early intervention [1,2].

Over the past decade, the aetiology of cardiometabolic conditions has been explored at increasing depths by ever larger genome-wide association studies (GWAS). However, many of the thousands of associated single nucleotide variants (SNVs) are outside of coding regions, and even for those that are, proving a causal implication of the corresponding protein is challenging using GWAS data alone. The exact causal cascade linking early phenotypes like inflammation and progression to disease therefore remains elusive.

To address this issue, protein quantitative trait loci (pQTL) studies have leveraged the genetics of circulating protein levels to discover causal relationships between dysregulated protein levels and disease phenotypes [[3], [4], [5]]. Coupled with causal inference and colocalisation analyses with other lines of evidence such as expression quantitative trait loci (eQTL) and drug target databases, such studies have demonstrated their potential of enabling the translation of GWAS findings into actionable targets.

Here, we perform an integrative whole genome sequence (WGS)-based pQTL discovery analysis for 92 proteins in blood serum samples of ∼3000 individuals in two isolated Greek populations [6]. The two distinct, isolated cohorts we investigated were selected for their unique cardiometabolic characteristics [7]. Moreover, genetic drift during extended isolation is known to increase the frequency of low-frequency and rare variants, enhancing statistical power for detection. These cohort characteristics offer a valuable perspective to study human complex traits, and in particular metabolic-related disease. Therefore, we measured proteins implicated in cardiometabolic traits within these cohorts to investigate the influence of genetic variation on these proteins that may act as intermediaries for cardiometabolic traits. The proteins in the Olink Cardiometabolic panel used in this study were selected by the manufacturer through text mining and manual curation for their role in cardiometabolic disease.

We find 322 pQTL loci, 76 of which are *cis-*acting. We describe in more detail genetic evidence for interaction between two *trans*-acting receptor–ligand pairs, as well as two interactions mediated by an unobserved protein. We show that several *trans* signals that can be unambiguously ascribed to a gene reflect interactions within inflammation and immune response pathways.

## 2 Materials and methods

### 2.1 Cohort information

The Hellenic Isolated Cohorts (HELIC) study is composed of two cohorts. The MANOLIS cohort is a collection of adult individuals from the Anogia and Mylopotamos villages from the mountainous regions of the Greek Island of Crete. The Pomak cohort is composed of adults from the Pomak villages, located in the mountainous mainland regions of Thrace, in North-eastern Greece. Participants in each cohort were required to have at least one parent from their village. Participants were primarily recruited through local medical centres, where blood sample for DNA extraction, lab-based haematological and biochemical profiling, and interview-based standard questionnaire were performed for each participant. The list of phenotypes available include biometric, anthropometric, and clinical evaluation measurements, biochemical and haematological profiles, and also self-reported medical history, demographic, socioeconomic, and lifestyle information. The MANOLIS cohort was named in honour of Manolis Giannakakis, 1978–2010. Both cohorts have been studied amply [6,[8], [9], [10], [11], [12], [13]] and have well-characterised diet profiles [7,14]. The study was approved by the Harokopio University Bioethics Committee and informed consent was obtained from every participant.

### 2.2 Sequencing and variant calling

500 ng of Genomic DNA was extracted from 1482 to 1642 samples for MANOLIS and Pomak, respectively. Standard Illumina paired-end DNA library was constructed using the extracted Genomic DNA according to the manufacturer's instructions. Constructed DNA libraries were subjected to 6 cycles of PCR amplification, followed by sequencing with the Illumina's HiSeqX platform at an average depth of 22.5× and 18.6× for MANOLIS and Pomak, respectively [4].

Basecall files for each lane were transformed into unmapped BAMs using Illumina2BAM, marking adaptor contamination and decoding barcodes for removal into BAM tags. PhiX control reads were mapped using BWA Backtrack and were used to remove spatial artefacts. Reads were converted to FASTQ and aligned using BWA MEM 0.7.8 to the hg38 reference (GRCh38) with decoys (HS38DH). The alignment was then merged into the master sample BAM file using Illumina2BAM MergeAlign. PCR and optical duplicates are marked using biobambam markduplicates and the files were archived in CRAM format.

Per-lane CRAMs were pooled on a per-sample basis across all lanes to produce library CRAMs; these were each divided in 200 chunks for parallelism. GVCFs were generated using HaplotypeCaller v.3.5 from the Genome Analysis Toolkit (GATK) for each chunk. All chunks were then merged at sample level, samples were then further combined in batches of 150 samples using GATK CombineGVCFs v.3.5. Variant calling was then performed on each batch using GATK GenotypeGVCFs v.3.5. The resulting variant callsets were then merged across all batches into a cohort-wide VCF file using bcftools concat.

### 2.3 Variant and sample quality control

Variant-level QC was performed using the Variant Quality Score Recalibration tool (VQSR) from the Genome Analysis Toolkit (GATK) v. 3.5-0-g36282e431, using a tranche threshold of 99.4% for SNPs, which provided an estimate false positive rate of 6%, and a true positive rate of 95%. For insertion or deletions (INDELs), we used the recommended threshold of 1%. For sample-level QC, we made extensive use of genotyping array datasets in overlapping samples, which provided sample matching information for 1,386 and 1,511 samples in MANOLIS and Pomak, respectively. In MANOLIS, a total of 25 individuals were excluded (n = 1457) based on sex checks, low concordance (<0.8) with chip data, duplicate checks, average depth (<10×), missingness (>0.5%), and contamination (Freemix or CHIPMIX score from the verifyBamID suite32 > 5%). This number was 27 for the Pomak cohort. In case of sample duplicates, the sample with highest quality metrics (depth, freemix and chipmix score) was kept. No samples were excluded in ORCADES.

### 2.4 Proteomics

92 protein levels for 1407 and 1610 samples from MANOLIS and Pomak, respectively, with whole-genome sequence data were measured. Measurements were made from 1 μL of serum using the Olink Target 96 Cardiometabolic (CMET) panel according to the manufacturer's instructions. Briefly, each protein assays are bound by antibodies labelled with unique DNA-oligonucleotides, which hybridise in pairs when in close-proximity. The hybridisation enables DNA polymerase to extend to create a DNA amplicon with a unique barcode to the protein assay. The amplicon is then quantified as Ct values by microfluidic real-time qPCR using the Fluidigm® Biomark instrument. To reduce potential technical variation, the Ct values of each analyte were normalised by subtracting against extension control, followed by another subtraction against the median of the inter-plate control values, and adjusting against a correction factor derived from negative controls. The final value is a Normalised Protein Expression (NPX) value, a relative quantification unit measurement of the assay with minimal variation within and across assay plates. Additionally, a lower limit of detection (LOD) value is determined for each protein based on the negative control signal plus three standard deviations. In this study, NPX values that fall below the LOD were included in the analysis.

In MANOLIS, two samples were excluded by the manufacturer's QC on all protein assays. One sample did not pass manufacturer QC for the CES1 protein. In Pomak, one sample was excluded in the ICAM3 protein assay, and 232 samples in ITGAM and DEFA1.

Missing ages were imputed by regressing all proteins that were non-missing in the samples without age on sex and age, and identifying those for which the coefficient P-value was lower than a Bonferroni-corrected threshold of 2 × 10^−4^. We then regress age on all these proteins and sex in all non-missing samples. For all NPX measurements that passed vendor QC, sex, age, age-squared, season of blood collection, assay plate number, and per-sample mean NPX level across CMET panel proteins were regressed out of the rank-based inverse-normal transformed measurements, followed by normalisation of residuals. Given the dry Mediterranean climate of Crete, we define season of collection as hot summer or mild winter. Plate effects are partially offset by the median-centering implemented by Olink. MANOLIS and Pomak samples were plated in the order of sample collection, which results in plate and season information to be largely correlated.

In total, 92 proteins were analysed in the present study, and the total sample size varied between 2783 and 3015 across both cohorts (Supplementary Table 1).

### 2.5 Single-point association and meta-analysis

Variants with either Hardy-Weinberg p ≤ 1 × 10^−5^ or missingness ≥1% were filtered out prior to association. We calculated an empirical relatedness matrix using GCTA v1.93.2 beta [15,16] after linkage disequilibrium (LD) pruning (parameters: window size = 50, step size = 5, and variance inflation factor = 2) and filtering out variants with MAF<5% using Plink v.1.9 [17]. Using the prepared input variant data and empirical relatedness matrix, we performed association using the MLMA linear mixed model algorithm of GCTA on both cohorts. QQ-plots were generated for 3 proteins with genomic inflation factor (λ_GC_)>1.05 (Supplementary Text, Supplementary Fig. 1). We use the 2011-03-25 release of METAL [18] for meta-analysis using inverse-variant based weighting. The genomic inflation factors across proteins had a mean of 0.996 post meta-analysis.

### 2.6 Significant signals and quality control

Previously, we used a stringent study-wide significance threshold of 7.45 × 10^−11^ computed on the effective number of traits and variants in our proteome analyses of the HELIC cohorts [4,13]. Here, we sought to verify this threshold through permutation testing. We first shuffled the sample column of the phenotype matrix in 100 random permutations. This results in 100 different genotype-phenotype pairs, where the genetic relatedness between samples and correlation between proteins is conserved. We used the same algorithm as above for association, with an empirical relatedness matrix calculated for every permutation. On average across the 100 runs, the proportion of variants below the customary genome-wide significance threshold of 5 × 10^−8^ was 1.56 × 10^−5^ for MANOLIS and 2.10 × 10^−5^ for Pomak, well below the expected 0.05. Although these results were generated in single cohort analyses, inverse-variance based meta-analysis of both cohorts would likely result in an even lower false-positive rate. This indicates that 5 × 10^−8^ is a conservative threshold in the current study, we therefore used it to declare study-wide significance in this work.

To aid analysis of signals, we merged individual-level data from the MANOLIS and Pomak cohort datasets with Plink 1.9, which implicitly excluded multiallelic variants. For each protein, we calculated the MAF equivalent to a minor allele count of 10 and filtered out variants with MAC<10 from each association summary statistic. We extracted significant loci from the resulting datasets using PeakPlotter v0.4.3 [19]. Briefly, PeakPlotter scans the summary statistics data and extracts all variants exceeding the significance threshold. It then produces locus boundaries and lead variants using a combination of LD-based clumping and merging of contiguous loci. The minimum width of a PeakPlotter association locus is 2 Mb, and loci closer to each other than 500 kbp are merged. A locus is defined as *cis*-acting if the gene coding for the protein is located within 500 kbp either side of the locus boundaries. Because pQTL association peaks can be very strongly associated and extend over many basepairs, we further examine signal loci located in proximity to each other, and merge them based on examination of regional association plots and LD between SNPs located at the respective locus boundaries. In this step, we also re-allocate *trans* loci as *cis* if there is evidence of residual association of a peak previously classified as *trans* over a *cis* locus.

### 2.7 Conditional analysis

We identify conditionally independent associated SNPs at a locus by performing conditional analysis using the conditional and joint association analysis (COJO) algorithm of GCTA (v1.93.2beta).

We first reduce LD at associated loci by clumping variants using Plink (parameters: clump-kb = 1000, clump-r2 = 0.05), removing clumps whose index variant P-value exceeded 5 × 10^−8^. We then performed conditional analysis on the filtered LD clump index variants for each signal locus using GCTA-COJO with the algorithm's default parameters. Significant independent variant signals were then collated for further analysis.

### 2.8 Functional annotation of conditionally associated SNPs

We assign a frequency category to each independent SNP as follows: common (MAF≥5%), low-frequency (1% ≤ MAF<5%), and rare (MAF<1%). Since rare variants are not the focus of this study, we do not consider signals where all independent variants belong to that category in downstream analyses. As part of peakplotter, rsIDs are extracted using the Ensembl REST API GET overlap/region endpoint, then matching alleles, and previous phenotype associations are retrieved using the phenotype/region endpoint, excluding COSMIC phenotypes. We extract consequences for all transcripts of protein-coding genes using the Ensembl REST API VEP endpoint, and mark variants whose consequence is equal to or more severe than missense according to Ensembl's calculated consequence list [https://www.ensembl.org/info/genome/variation/prediction/predicted_data.html]. We extract variants with LD r^2^ > 0.8 to any independent hit within a 2 Mb window, and perform the same analysis, marking those which tag severe variants.

### 2.9 Mapped gene

We further attempt to assign signal-to-gene relationships. For *cis* signals, we map independent variants to the gene coding for the protein. For *trans* signals, we first identify loci that map to known pleiotropic or master regulator genes (ABO, FUT2, KLKB1, F12). None of the remaining independent *trans* hits were, or were tagging, a high-consequence variant for a protein-coding gene. We therefore assigned a variant to a gene if it conditionally co-localised with a *cis-*eQTL for that gene (see eQTL colocalisation).

### 2.10 Novelty analysis

To examine novelty of our signals, we pooled summary statistics from 46 studies which had previously analysed circulating levels of the proteins present on our panel (Supplementary Table 2). We collect information about previously established signals from the primary and supplementary tables in these articles, and merge it to produce a database of 9,242,846 previous associations. We collect study information such as author, PMID, ethnicity and size of discovery cohort, and peak information, including UniProt ID, coordinates, alleles, allele frequencies, effect sizes and direction, mapped gene, p-value, and *cis*/*trans* status. We manually map 12 archived rsIDs to their current counterparts. Where build 38 position information was not available, we fetched positions corresponding to the rsIDs using the Ensembl REST API Variation endpoint. For variants which have build 37 position info but no rsID, a lift-over was performed using the Ensembl REST API Map endpoint. Some associations did not report UniProt IDs. For those that reported gene symbols, we first convert gene symbols to Ensembl stable IDs using the REST API ID endpoint, then convert those to UniProt IDs using the EMBL-EBI Proteins API [20]. Finally, we manually investigate 204 protein names which were not resolved this way. 64 of them did not correspond to any UniProt ID and the corresponding fields were set to missing. We verify that this set of previous associations contains all associations with protein levels retrieved via the GWAS catalog.

We investigate both locus-level and variant-level novelty. We call a locus novel if no association with the same protein overlaps the peak boundaries as defined by PeakPlotter (minimum 2 Mbp wide). We call a variant novel if it is not in LD (>0.8) with any variant that has been previously associated with the same protein within a 2 Mb window.

### 2.11 Mendelian randomisation analysis

The significant independent variant signals were subjected to two-sample Mendelian randomisation (2SMR) analysis. Given the broad cardiometabolic focus of the assayed proteins, we focused on a list of cardiovascular and metabolic-related traits: heart attack, cardiovascular disease and atherosclerosis, stroke, type-2 diabetes, hyperlipidemia, dyslipidemia, obesity, metabolic syndrome, hypertension, insulin resistance, hyperinsulinemia, hyperleptinemia, non-alcoholic fatty liver disease, chronic renal failure and nephropathy. The list of corresponding studies is given in Supplementary Table 3.

We perform 2SMR analysis using the R programming language (v4.1.1) with the TwoSampleMR (v0.5.6) [21,22] package. For each disease and associated continuous traits above, we first check whether a large, recent GWAS study is available in the MRC IEU OpenGWAS database [23]. For those that do, we download the outcome data using the convenience function provided by the TwoSampleMR package. For summary statistics that are not available on the MRC IEU OpenGWAS database, we download the raw summary statistics and reformat into an outcome dataset. For raw summary statistics that have no rsID information, we extract the rsID for each variant using Ensembl REST.

TwoSampleMR assumes that the instruments of the exposure data are independent of one another. Moreover, TwoSampleMR matches instruments in the exposure data to the outcome data based on rsIDs. Therefore, for each independent variant signal, we collect all variants with LD > 0.8 in the merged HELIC dataset within the signal boundaries, and query the Ensembl REST API GET overlap/region endpoint to extract the rsID for each variant in LD based on position and allele information. All INDEL variants are excluded during this process, as no INDEL variant exactly matches the position and allele information of rsID variant queried from Ensembl. The exposure and outcome data are merged using the TwoSampleMR harmonise_data function, and for each independent LD-clump, the rsID-bearing SNP with the strongest meta-analysis p-value is selected as the instrument of the exposure data. Tagging instruments for all the independent signals are then merged across all loci per protein for *cis* and *trans* instruments, and across *cis* regions for *cis*-only instruments. For *trans* loci, we exclude signals corresponding to master regulator or pleiotropic genes form all MR analyses.

### 2.12 PheWAS

All variants with an rsID and in LD > 0.8 with independent signals are queried against the GWAS Catalog's all-associations file release 1.0.2.20220411 and PhenoScanner V2 [24] to collect all previously associated phenotype signals for each independent pQTL signals. From the PhenoScanner query results, we exclude signals from the Neale Lab UK Biobank (UKBB) data.

### 2.13 Colocalisation analysis

We perform Bayesian-based colocalisation analysis using fast-coloc [https://github.com/hmgu-itg/fast.coloc] between our pQTL signals and both eQTL and cardiometabolic complex trait GWAS datasets.

For colocalisation against eQTL, we use the publicly available GTEx v8 summary statistics for all 54 tissue types [25] [https://gtexportal.org/home/datasets]. For both *cis* and *trans*-pQTL signals, we extract eQTL data for all protein coding genes within a +/−500 Kb window. Effect allele frequency (EAF) information is not reported in GTEx summary statistics. We therefore match the MAF reported by GTEx with the alternate allele frequency reported by the 1000 Genomes Project Phase 3 data [26]. Allele frequencies can differ between 1000 Genomes and GTEx, and matching can be ambiguous for variants whose MAF is close to 0.5. We therefore perform two 2-sample chi-square tests for equality of proportions, one for the case where the minor allele is the reference, one where it is the alternate. To select between these cases, we compute the difference of the corresponding chi-square statistics, which follows a variance-gamma distribution. We compute quantiles and p-values using the VarianceGamma package available in R, using ν = 2, c = 0, θ = 0, σ = 2. We consider any position where the resulting p-value is smaller than 0.05 to be unmatchable. We also use an empirical hard threshold of 5 × 10^−10^ for the smallest binomial p-value, to exclude cases where the best allele is decidable, but the difference in allele frequencies between GTEx and the reference is too high.

Fast-coloc assumes that the given summary statistic contains a single independent signal variant. For eQTL summary statistics, we perform stepwise model selection with GCTA-COJO using the 1000 Genome Project Phase 3 data as a reference sample to identify independent signals. If multiple independent signals are detected, each independent signal is subjected to “leave-one-out” conditional analysis using GCTA-COJO.

For complex traits, we use PhenoScanner V2^24^ to query all available GWAS signals at each pQTL signal locus. We exclude Neale Lab UKBB data from the query results, and filter for cardiometabolic-relevant trait signals in European and/or Mixed-ancestry studies published in 2015 and onwards. All signals are then grouped according to their study, trait, and ancestry to create a summary statistic for each group. The PhenoScanner database does not store the EAF of the signal variant in the studied cohort. Therefore, depending on the ancestry of the studied cohort, we assign the 1000 Genome Project ancestry-specific allele frequency (EUR or Mixed) of the effect allele as the signal's EAF. At the time of querying, the latest study included in the PhenoScanner database was from 2018. We also perform colocalisation analysis with all the summary statistics used in MR (Supplementary Table 3). We detect independent signals using GCTA-COJO in each summary statistics file, and perform “leave-one-out” conditional analysis if necessary.

Once all eQTL and complex trait summary statistics were prepared with each only containing one independent signal, we perform colocalisation for all pQTL locus on all selected eQTL and overlapping complex traits.

## 3 Results

We find 322 loci associated at genome-wide significance (Supplementary Fig. 2), involving 455 independently associated variants (Supplementary Table 4). 76 loci are *cis*-acting and 246 are *trans*-acting (Supplementary Figs. 3–4). 187 of the independently associated variants are common (minor allele frequency (MAF) ≥0.05), 72 are low-frequency (0.01 ≤ MAF<0.05) and 196 are rare (MAF<0.01).

210 signals involving 218 independent variants have no prior evidence of association at the same locus for the same protein in previous pQTL studies (see Methods). We also report the association between 108 variants and protein levels at 26 known loci that are conditionally independent of all previously described variants associated with the same protein in the region.

62 *cis*-acting and 64 *trans*-acting signals co-localise with GTEx [27] eQTL in at least one tissue (Supplementary Table 5), and in 12 and 16 cases, a colocalisation is specifically observed in whole blood. Among these *cis*, 15 (24.19%), 23 (37.10%), and 24 (38.71%) co-localise with only *cis*, only *trans*, and both *cis* and *trans* eQTLs, respectively. Interpretation of *trans* signals colocalising with *trans*-eQTL signals should be made with caution, as there remains a possibility of a third factor (e.g. transcription factor or gene regulatory components) that drives the shared signal instead of a direct causation between the protein and gene.

77 signals across 59 proteins show significant colocalisation with at least one cardiometabolic trait GWAS (Supplementary Table 6), highlighting the intermediate role of many proteins in disease-relevant phenotypes. To specifically examine causal relationships between dysregulated protein levels and disease, we perform Mendelian randomisation (MR) analysis using both *cis*-pQTLs only and *cis* and *trans*-pQTLs against a range of relevant clinical outcomes (Supplementary Table 7). 56 proteins show causal association with at least one clinically relevant trait in *cis*, including type 2 diabetes, blood pressure, and osteoarthritis. When *trans* loci are included, 10 causal signals are attenuated, while 15 further proteins show evidence of causation. We note that the interpretation of these fluctuations, and of *trans*-mediated MR signals in general, is challenging due to the possibility of protein/complex-trait pleiotropy at these loci.

**Figure 1:**
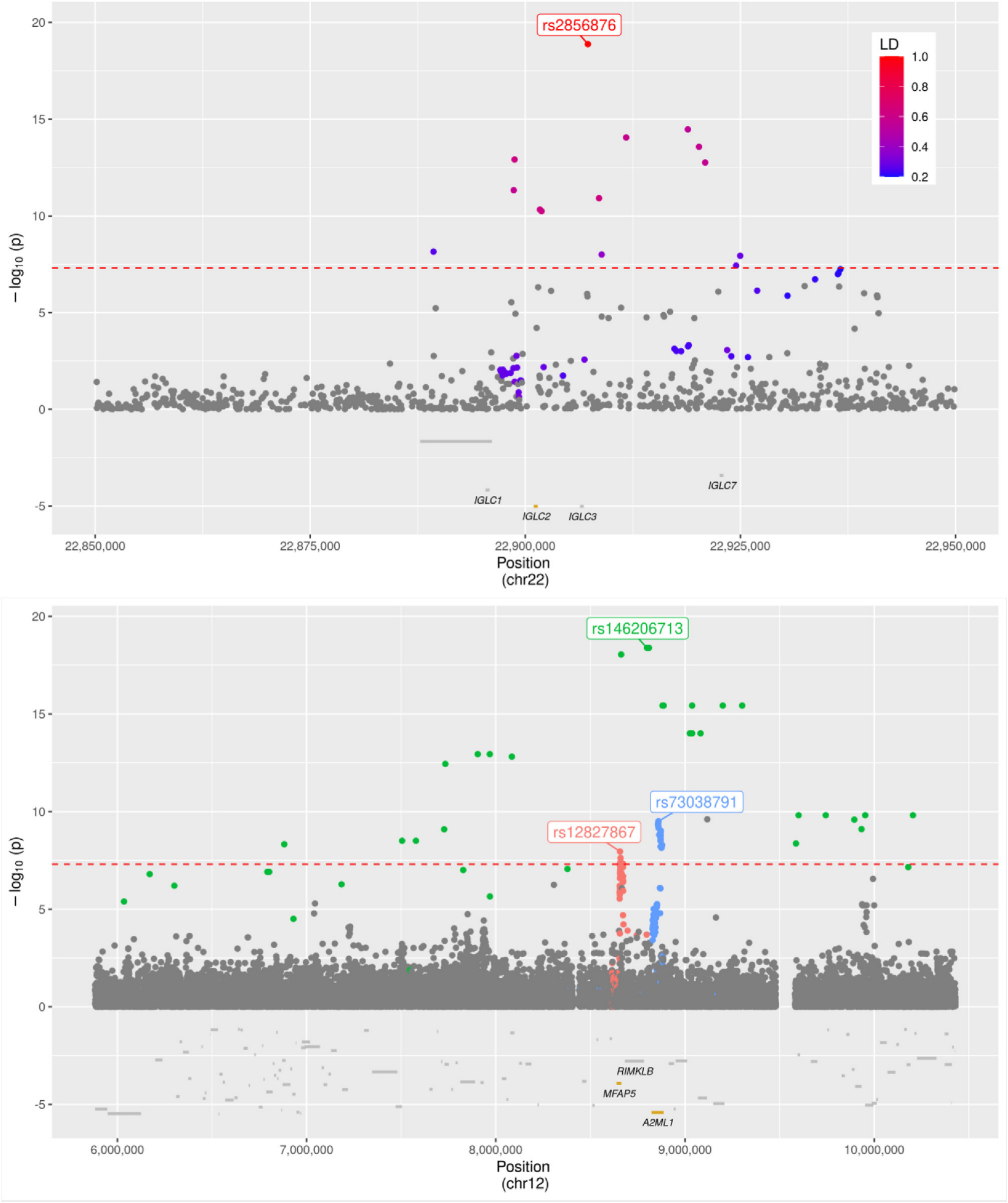
**Regional associations around the IGLC2(top) and MFAP5(bottom) *cis* loci.** For IGLC2, only one variant contributes to the locus, so a LD colour scale is used. For MFAP5, the LD blocks (LD > 0.2) corresponding to the three independent signals are displayed in a separate colour.

We describe in detail two *cis-*associations (Figure 1). The first is led by a common intergenic variant (rs2856876, β = 0.3202, σ = 0.0354, p = 1.33x^−19^, MAF = 0.1751) downstream of *IGLC2*, the *cis* gene coding for Immunoglobulin lambda light chain constant 2, and colocalises with an eQTL for that gene and also with 4 other *IGLC* genes (Supplementary Text). Three variants contribute to a second *cis-*association influencing levels of Microfibrillar-associated protein 5 (MFAP5), the common intronic rs12827867 (MAF = 0.1252, β = 0.2239, σ = 0.0392, p = 1.11 × 10^−8^), the rare intergenic rs146206713 (MAF = 0.0065, β = −1.831, σ = 0.205, p = 4.14 × 10^−19^), and the *A2ML1* intronic rs73038791 (MAF = 0.0783, β = 0.3144, σ = 0.05, p = 3.19 × 10^−10^). *MFAP5* loss-of-function has been associated with increased risk of Marfan-negative familial thoracic aortic aneurysm (TAA) [28], and we find a causal relationship between these MFAP5-altering variants and an increase in blood pressure. Hypertension is highly prevalent in TAA [29].

We identify 16 common and low-frequency novel *trans* loci where the lead variant shows strong evidence of association (p < 7.45 × 10^−11^). (Supplementary Text, Table 1). We discuss three of these signals in more detail here.

**Table 1:** *trans* – acting pQTL described in the present study.

protein	Mapped gene	position	rsID	consequence	closest or consequence gene	MAF	beta	se	p
CCL5	*ACKR1*	chr1:159205704	rs34599082	missense	*ACKR1*	0.0674	0.719	0.0682	5.22 × 10^−26^
		chr1:159205564	rs12075	missense	*ACKR1*	0.4226	0.271	0.027	9.15 × 10^−24^
		chr1:159204893	rs2814778	5′UTR	*ACKR1*	0.0144	−0.794	0.115	5.96 × 10^−12^
GAS6	*AXL*	chr19:41233275	rs66841352	intronic	*AXL*	0.3763	−0.2805	0.0278	5.57 × 10^−24^
LYVE1	*GCNT1*	chr9:76503484	rs147866228	missense	*GCNT1*	0.0491	−0.563	0.0746	4.31 × 10^−14^
GP1BA	*TIMP3*	chr22:32765566	rs1079734	intronic	*SYN3*	0.3905	0.2005	0.0278	5.68 × 10^−13^
TGFBR3	*KNG1*	chr3:186742138	rs710446	missense	*KNG1*	0.4972	0.194	0.027	6.54 × 10^−13^
TIE1	*unknown*	chr16:63021	rs370013567	missense	*RHBDF1*	0.0131	−1.14	0.163	2.2 × 10^−12^
CCL5	*unknown*	chr10:102113782	rs115703265	intronic	*LDB1*	0.0474	0.6053	0.0888	9.57 × 10^−12^
TIMP1	*TENT5C*	chr1:117585523	rs320366	intergenic	*TENT5C*	0.3501	−0.192	0.0287	2.26 × 10^−11^
TGFBI	*ORM1/ORM2*	chr9:114321523	rs150611042	regulatory region	*ORM1*	0.068	−0.362	0.0551	4.69 × 10^−11^
LCN2	*CFH*	chr1:196727803	rs1410996	Intronic	*CFH*	0.4349	−0.178	0.0274	6.91 × 10^−11^

Three independent *ACKR1* variants are associated with levels of the chemotactic C–C Motif Chemokine Ligand 5 (CCL5): the common missense rs34599082 (MAF = 0.0674, β = 0.719, σ = 0.0682, p = 5.23 × 10^−26^), the common missense rs12075 (MAF = 0.4226, β = 0.2712, σ = 0.027, p = 9.16 × 10^−24^), and the low-frequency 5′ UTR variant rs2814778 (MAF = 0.0144, β = −0.794, σ = 0.1154, p = 5.96 × 10^−12^). CCL5 was shown to be an ACKR1 ligand in competitive ligand studies [30], yet this is the first time this link has been shown through genetic evidence. *ACKR1* is also known as DARC, the Duffy antigen receptor for chemokines, and rs12075 and rs2814778 determine two of the Duffy blood groups [31]. The protein is expressed mainly on erythrocytes, but is also found in endothelial cells, kidney duct epithelial cells, as well as in the lung alveolae, thyroid, colon and spleen [32].

The common intronic *AXL* variant rs66841352 is associated with reduced levels of Growth Arrest Specific 6 (GAS6) protein (MAF = 0.3763, β = −0.2805, σ = 0.0278, p = 5.57 × 10^−24^). AXL is a ubiquitously expressed member of the TAM (TYRO3, AXL, MER) family of receptor tyrosine kinases. Similar to ACKR1/CCL5, AXL/GAS6 are an experimentally proven receptor–ligand pair [33,34]. We also observe a weaker reverse association, where the *GAS6* intronic rs142867480 (MAF = 0.185, β = 0.1797, σ = 0.0344, p = 1.78 × 10^−7^) increases the levels of AXL, and is in strong LD with variants implicated in glycated hemoglobin, triglyceride and LDL levels. AXL activation is thought to be the main purpose of GAS6 [35].

**Figure 2:**
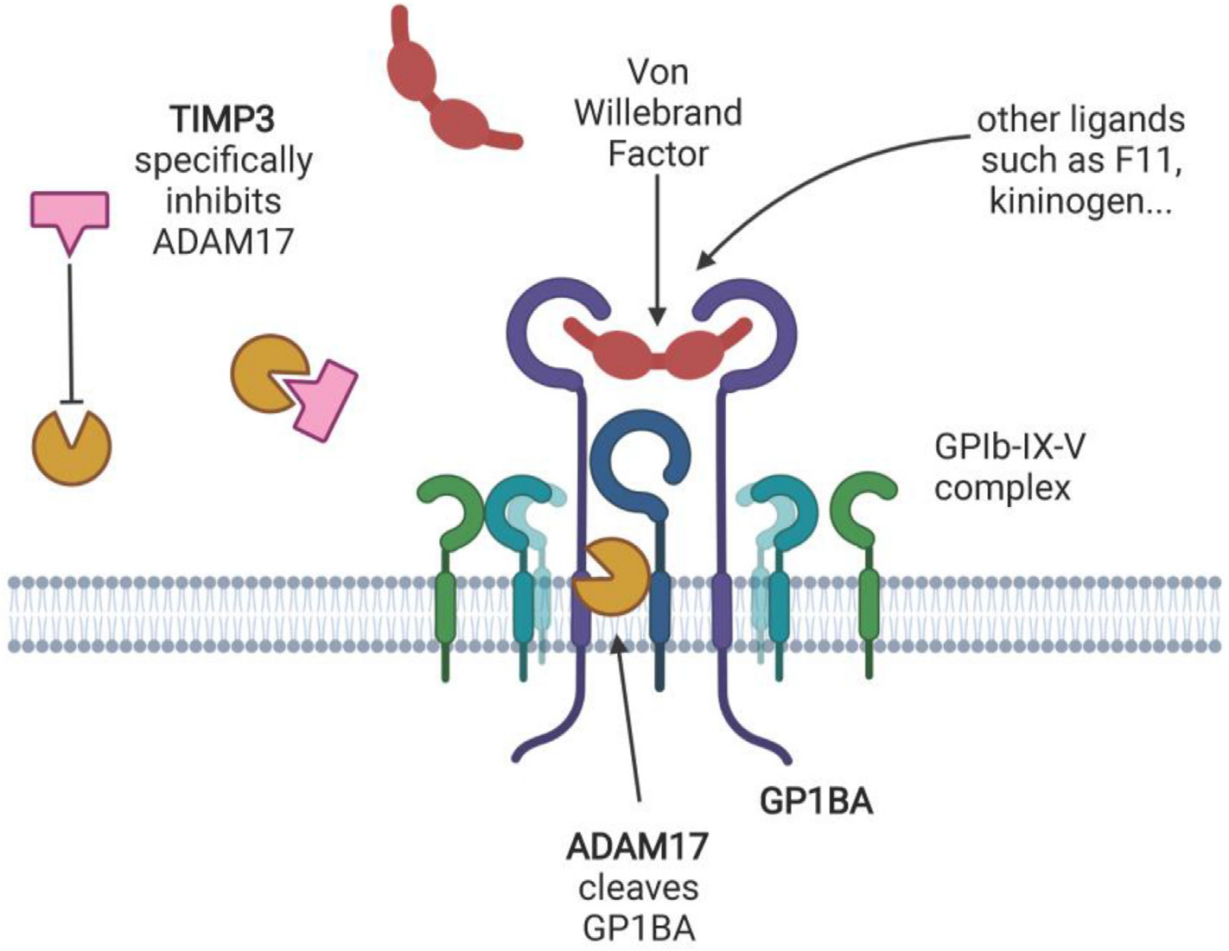
**Proposed mechanism underlying the GP1BA association at the TIMP3 locus.** GP1BA (purple hook) is shown as a part of the fully-formed GP1B-IX-V complex on the platelet surface with VWF, one of its major ligands (red). ADAM17 (brown) is shown cleaving GP1BA from the cell surface as part of receptor shedding. TIMP3 (pink) specifically inhibits ADAM17. Created using BioRender.

The common *SYN3* intronic variant rs1079734 (MAF = 0.3905, β = 0.2005, σ = 0.0278, p = 5.68 × 10^−13^) is associated with increased levels of Platelet glycoprotein Ib alpha chain (GP1BA). This protein binds to GP1BB to form glycoprotein (GP) Ib, which in turn combines with other GP subunits to form the GPIb-IX-V complex. This complex is found exclusively on the surface of platelets, and primarily binds von Willebrand factor (VWF), although it has other ligands, such as thrombin, P-selectin, F11 and F12. Binding of this complex to VWF plays a key role in coagulation, particularly platelet activation and adhesion upon damage to vasculature or shear stress. It is also involved in several other homeostatic and disease processes such as thrombosis, stroke and myocardial infarction. Our signal colocalises with eQTL for the nearby *TIMP3* gene but not *SYN3,* in multiple tissues with concordant direction of effect, and the same SNV has been reported as a TIMP3-decreasing pQTL [36]. TIMP3 is a tissue inhibitor of matrix metalloproteinases which specifically inhibits ADAM17, both being released upon platelet activation [37]. In turn, ADAM17 cleaves platelet GP1BA as part of metalloproteolytic receptor shedding, a key modulator of platelet reactivity and adhesion [38]. This signal therefore likely reflects modulation of GPIb-V–IX shedding in response to genetically perturbed TIMP3 RNA and protein expression (Figure 2).

## 4 Discussion

In this study, we combine whole genome sequencing with proteomic data to discover pQTLs and link these to clinically-relevant traits. Those pQTLs described in this work that could unambiguously be ascribed to a gene are involved in multiple overlapping pathways, such as inflammation and immune response (Supplementary Text).

Several of the interactions described in this manuscript involve proteins that are either dysregulated in cardiometabolic disease or actively being investigated as therapeutic targets. For example, the AXL/GAS6 pathway is generally associated with conditions of injury, inflammation, and repair, and signaling is downregulated in multiple chronic inflammation and autoimmune disorders [39]. Over 50 drugs targeting this system have been developed, both in the form of signaling inhibitors and activators such as recombinant GAS6 for the treatment of MS [40]. The axis has been described as an important pathogenic mechanism for cardiovascular and renal complications associated with diabetes [41], and GAS6 is dysregulated in type 2 diabetes (T2D) [42]. Similarly, for the CCL5/ACKR1 receptor/ligand pair, one of the conditioned signals (rs12075) colocalises with a GWAS hit for glycated hemoglobin with consistent direction of effect (Supplementary Table 6). CCL5 directly affects insulin signalling [43], and aggravates inflammatory responses in adipocytes, causing insulin resistance and obesity [44].

For *trans* signals that do not reflect receptor/ligand interaction, one or both of the interactants can be disease-relevant, which can reflect either mediation, regulation or co-activation. For example, in the LCN2/CFH interaction, expression of the former is observed in kidney and liver injury, lung inflammation and Inflammatory bowel disease-positive intestinal epithelium. Circulating LCN2 is positively correlated with adiposity, triglyceridemia, insulin resistance, and obesity-related metabolic disorders, as well as heart failure and renal syndrome [45,46]. These associations may be explained by LCN2's fatty acid-binding capacity, in addition to its role in the inflammation prevalent in those conditions [47]. As a target, LCN2 is being investigated for brain injury [48], and is a potential biomarker for various cardiometabolic disorders [46]. The LCN2-decreasing *CFH* variants reported here were found by MR to causally reduce blood pressure, body mass index and waist-to-hip ratio, but increase risk of stroke, triglycerides and LDL levels. Furthermore, the LCN2 signal also colocalises with eQTL signals of nearby *CFH* genes. However, given the inherent pleiotropy of *CFH* variants, evidence of causality regarding LCN2 in particular should be taken with caution. The present study did not discover any LCN2 *cis*-pQTL that would have allowed to test for the direct causal effects of LCN2 on cardiometabolic disease.

*Cis*-signals are easier to interpret. For example, *IGLC2* encodes one of the constant subunits of immunoglobulin light chains. These can exist as free light chains, in its homodimerized form, or as part of antibody complexes. Free light chains, once considered a byproduct of Ig synthesis, have recently been investigated as biomarkers for inflammation and T2D [49] as well as diabetic kidney disease [50]. For the second *cis* signal, clinical studies have linked MFAP5 levels with obesity-associated inflammation [51]. MFAP5 is overexpressed in diabetic mouse models, and diabetes-induced cartilage degeneration is decreased in gene knockouts [52].

A protein-coding SNV may affect antibody binding rather than true protein abundance. This would lead to measurement error by antibody-reliant assays such as the one used in this study, a phenomenon known as epitope effects. Protein-coding *cis*-pQTL signals should be interpreted with caution, and preferably supported with orthogonal evidence such as eQTL colocalisation with concordant effect direction. The two newly reported *cis* loci are both supported by eQTL evidence (Supplementary Table 6).

In this work, we describe a WGS-based association study of 92 serum protein levels in two European population isolates. The proteins under study were curated for their cardiometabolic relevance through text mining and literature searches, which we confirm through a wide observed overlap of pQTLs and associations for complex cardiometabolic traits. The genetic associations we have identified, both in *cis* and *trans,* involve inflammatory pathway genes, providing genetic support to existing clinical links between chronic inflammation and cardiometabolic diseases.

## Author contributions

Analysis: AG, YCP.

Phenotype collection: ET, MK, GD.

Supervision: EZ.

Manuscript writing: AG, YCP, EZ.

## Declaration of Competing Interest

All authors declare no conflict of interest.

## Data Availability

The MANOLIS sequencing data used in this study are available at the European Genome-Phenome Archive (EGA) under accession number EGAS00001001207.
