# Supplementary material for: Genome-wide meta-analysis of 92 cardiometabolic protein serum levels
Source: Mol Metab. 2023 Sep 29;78:101810. doi: 10.1016/j.molmet.2023.101810 (PMC10582065; doi:10.1016/j.molmet.2023.101810)
Supplement: Multimedia component 9 [file mmc9.pdf]

Supplementary Text

Proteins with Genomic inflation factor (λ_{GC})>1.05

CD46 and C2 in Pomak, and QPCT in MANOLIS cohort exhibited λ_{GC}>1.05. Despite these values, these proteins were not excluded from further analyses. Several factors can explain for a λ_{GC}>1.05, such as population stratification or a high degree of polygenicity. The former is not a concern for these genetically homogeneous populations. Instead, a slight inflation in the λ_{GC} value can be ascribed to increased power and abundance of signal. This can be evidenced by inspecting the QQ-plots, where the expectation is that the inflation is not seen across the distribution but rather at the tail of the distribution (Supplementary Figure 1).

Novel *cis* signals

IGLC2

IGLC2 codes for the constant part of the lambda light chain of immunoglobulins, which are archetypal inflammatory and immune response proteins (Supplementary Figure 5). The association is led by a common intergenic variant (rs2856876, beta=0.3202, se=0.0354, $p=1.33x10^{-19}$, MAF=0.1751) downstream of *IGLC2*. This variant was previously associated with increased levels of the FOXJ2 and SCP2D1 proteins[1] (Supplementary Table 8). The former is a widely expressed transcription factor, the latter a sterol binding protein involved in intracellular cholesterol transport and steroid biosynthesis. The association colocalized with eQTL for *IGLC1, IGLC2, IGLC3, IGLC6,* and *IGLC7* in multiple tissues. In addition to this *cis* locus, we find two previously undescribed *trans* loci for IGLC2. The single low-frequency *ALDH7A1* intronic rs1037232792 (β=-0.745, σ=0.131, $p=1.32x10^{-8}$) is present at extremely low frequencies in cosmopolitan populations ($MAF=7.96x10^{-6}$ in TOPMed), yet it is enriched to a MAF of 0.012 and 0.00539 in Pomak and MANOLIS, respectively. This gene encodes an aldehyde dehydrogenase that degrades aldehydes generated by alcohol metabolism, lipid peroxidation, and other forms of oxidative stress. The low-frequency *RPIA* intronic indel rs555703356 (MAF=0.0171, β=-0.636, σ=0.113, $p=1.68x10^{-8}$) leads an association peak that extends over *EIF2AK3*. This signal co-localises with eQTL for *RPIA* in multiple tissues including whole blood with consistent direction of effect, and with an *EIF3AK3* eQTL in non-sun-exposed skin (Supplementary Table 5). *RPIA* encodes Ribose 5-phosphate isomerase A, a member of the pentose phosphate pathway which takes part in glucose metabolism, and which has been implicated in diabetes[2].

MFAP5

MFAP5 encodes an ECM protein that reacts to pro-inflammatory cytokines[3] and promotes the expression of matrix metalloproteinases[4], which are important regulators of inflammatory processes[5] (Supplementary Figure 5). We report a *cis*-MFAP5 signal where two variants contribute independently according to conditional and joint association: the common intronic rs12827867 (MAF=0.1252, β=0.2239, σ=0.0392, $p=1.11x10^{-8}$) and the rare intergenic rs146206713 (MAF=0.0065, β=-1.831, σ=0.205, $p=4.14x10^{-19}$). rs12827867 tags the splice region rs12833793 and the 5' UTR rs71447527, and this signal colocalizes with *MFAP5* eQTL in spleen with concordant direction of effect. A third signal is present, led by the *A2ML1* intronic rs73038791 (MAF=0.0783, β=0.3144, σ=0.05, $p=3.19x10^{-10}$), however this signal gets attenuated to $p=5.03x10^{-5}$ after conditioning for both other signals, despite low LD (r^2=0.050 and $9.03x10^{-3}$ with rs146206713 and rs12827867, respectively. *MFAP5* encodes microfibril-associated protein 5, a component of the microfibrils of the extracellular matrix highly

expressed in adipose tissue. Its expression correlates with markers of insulin resistance in metabolic syndrome[6]. *MFAP5* loss-of-function has been associated with increased risk of Marfan-negative familial thoracic aortic aneurysm (TAA)[7], and we find a causal relationship between these MFAP5-decreasing variants and an increase in blood pressure. Our MFAP5-associated variants are not nominally associated in a recent GWAS of TAA[8].

Novel *trans* signals not discussed in the main text

CCL5 *trans ACKR1*

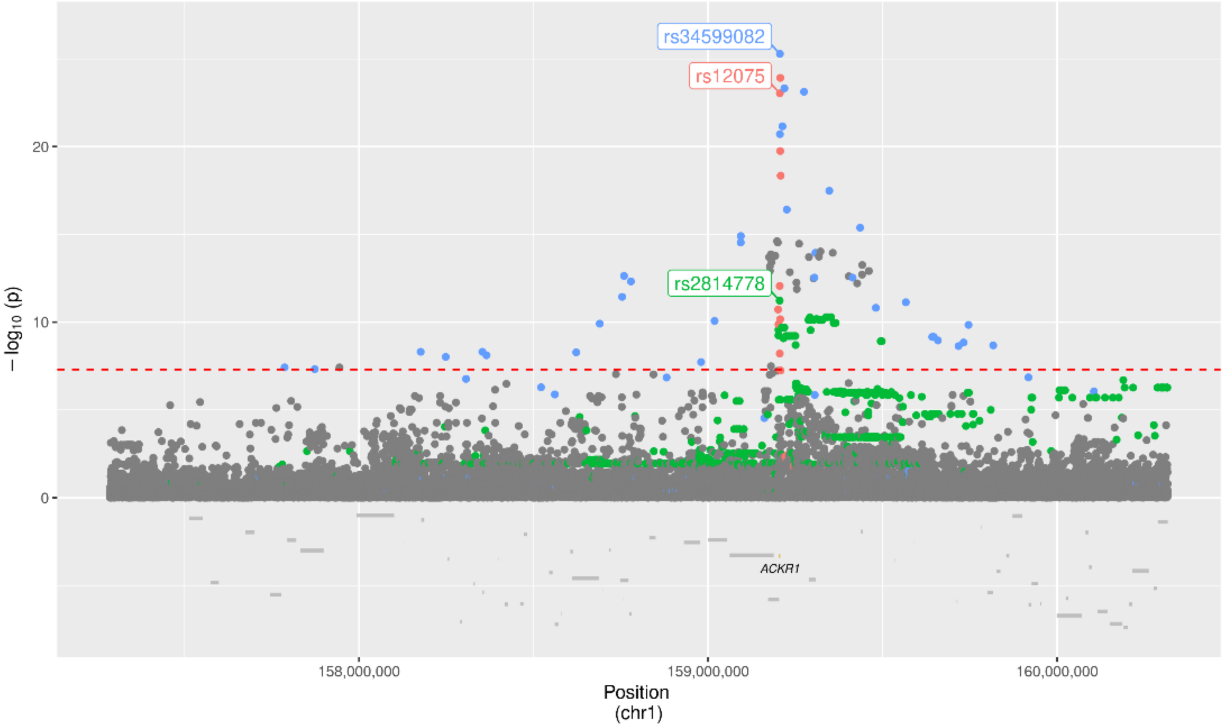

CCL5 is a proinflammatory chemokine expressed by T-cells, monocytes, epithelial cells, fibroblasts and thrombocytes, that recruits leukocytes to the site of inflammation. ACKR1 readily binds other pro-inflammatory CCL and CXCL chemokines, and can act as a chemokine sink on erythrocytes[9]. Scavenging by ACKR1, as indicated by the CCL5/*ACKR1 trans*-pQTLs in the main text, likely plays an important role in controlling the localization and intensity of the inflammatory response. This pro-inflammatory gradient affects numerous systems, including the extracellular matrix. However, ACKR1 has been shown to be functionally redundant in mice[10], which is thought to be a common phenomenon in the chemokine system, as evidenced in humans with the redundance of the CCR5 receptor[11]. ACKR1 being the point of entry of *plasmodium* malarial parasites, expression of this gene is thought to have been selected against in regions where the disease was endemic, explaining the high prevalence of Duffy-silent phenotypes in individuals of African descent.

LCN2 *trans CFH*

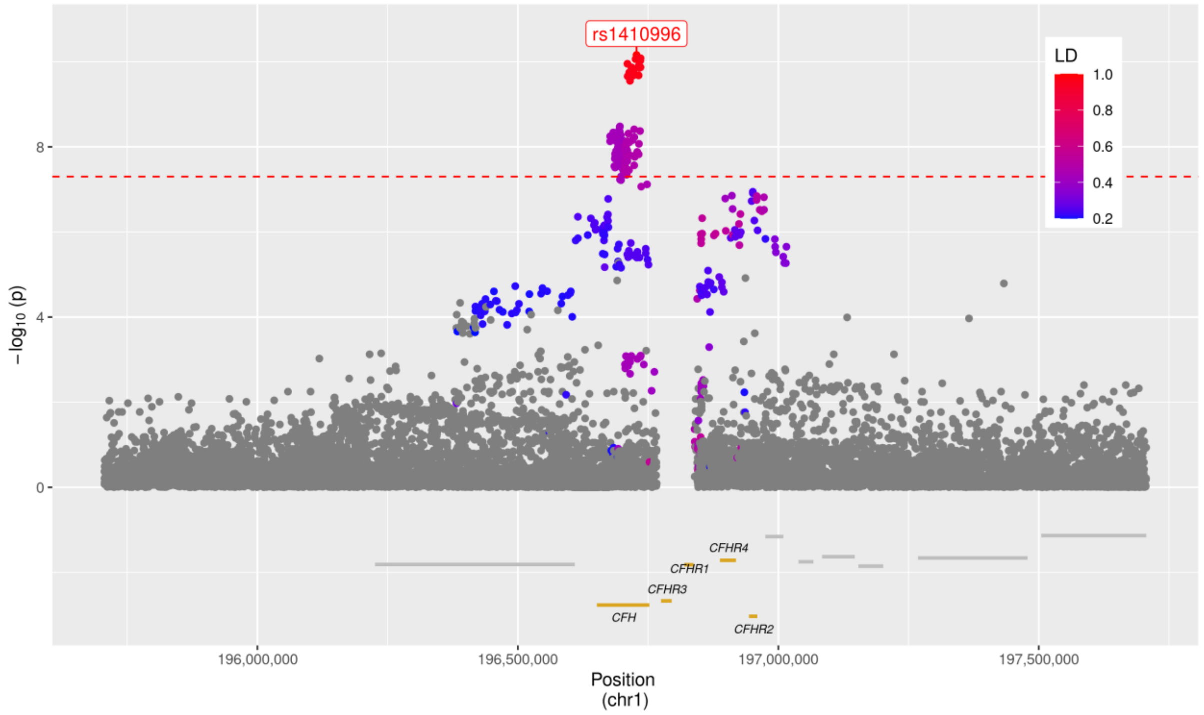

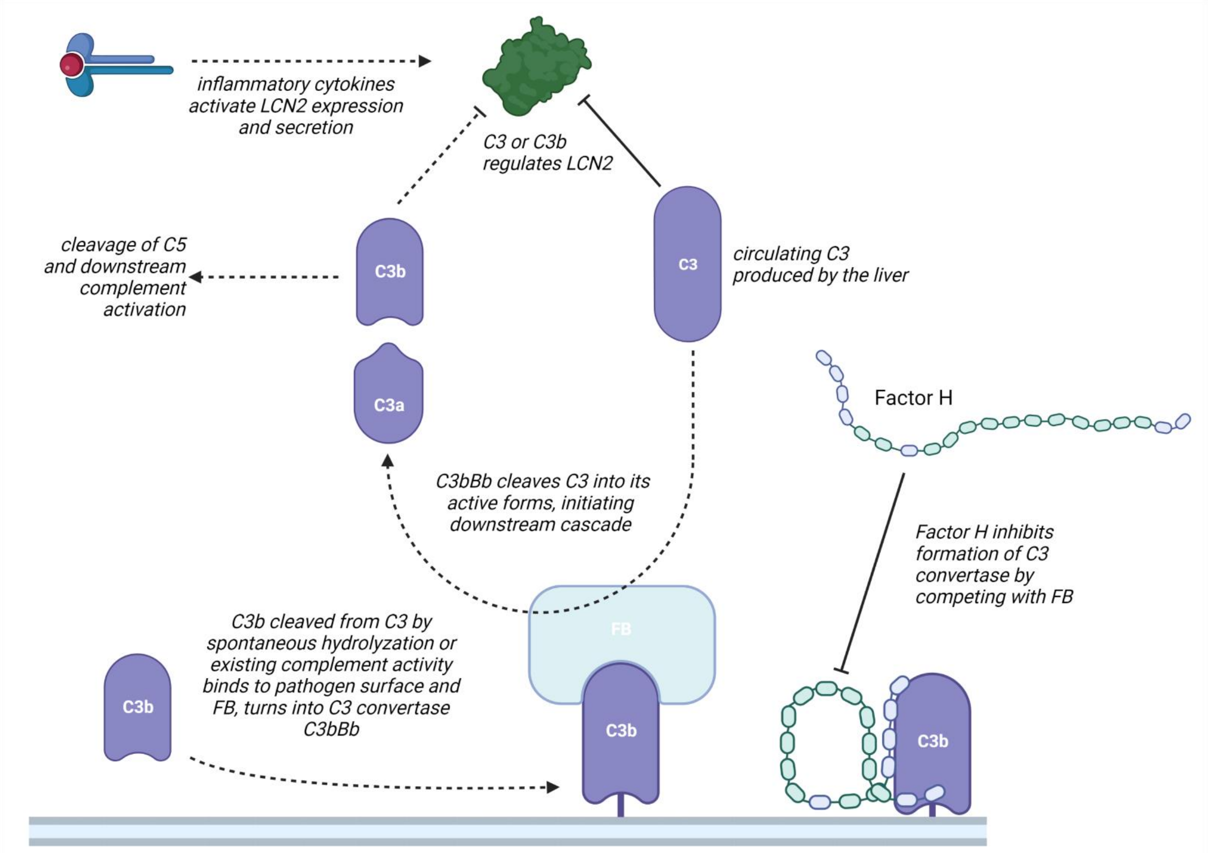

The *CFH* intronic rs1410996 is associated with decreased levels of lipocalin-2 (MAF=0.4349, β=-0.1788, σ=0.0274, p=6.91x10^{-11}). Although the signal is located within *CFH*, it only colocalises with eQTL for neigbouring *CFHR* genes in multiple tissues, although it is in strong LD with CFH pQTL variants. CFH encodes complement factor H, a main modulator of the alternative complement pathway in innate immunity, through its regulatory effect on C3. *CFHR* genes code for various receptors that compete with CFH ligands, therefore having a pro-inflammatory effect. Due to the central immunomodulator

role of these proteins, CFH locus variants have been implicated in many traits and diseases. Lipocalin 2 is regulated by C3, the main target of CFH[12,13].

Lipocalin-2 was originally described as an important factor in innate immunity and bacterial defense[14]. It blocks bacterial siderophores from acquiring iron[15] and promotes inflammation[16,17]. It is believed to be a more versatile, neutrophil modulating and chemotactic agent of immunity and stress response, similar to other granule markers like myeloperoxidase[18].

LYVE1 *trans GCNT1* missense

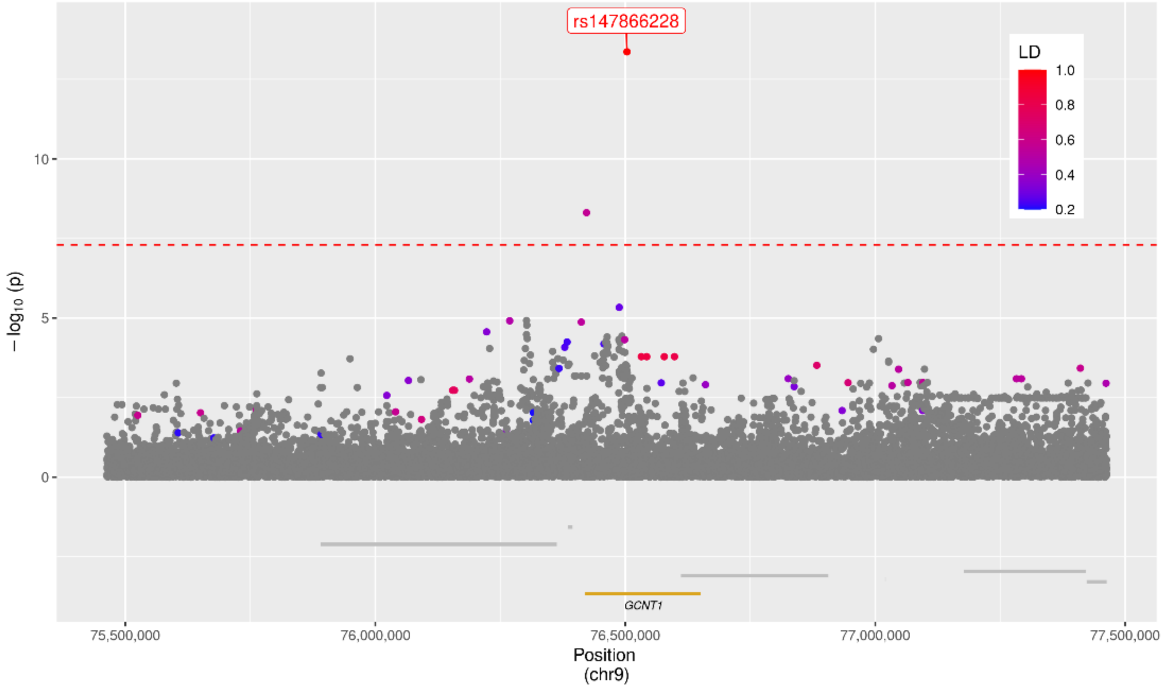

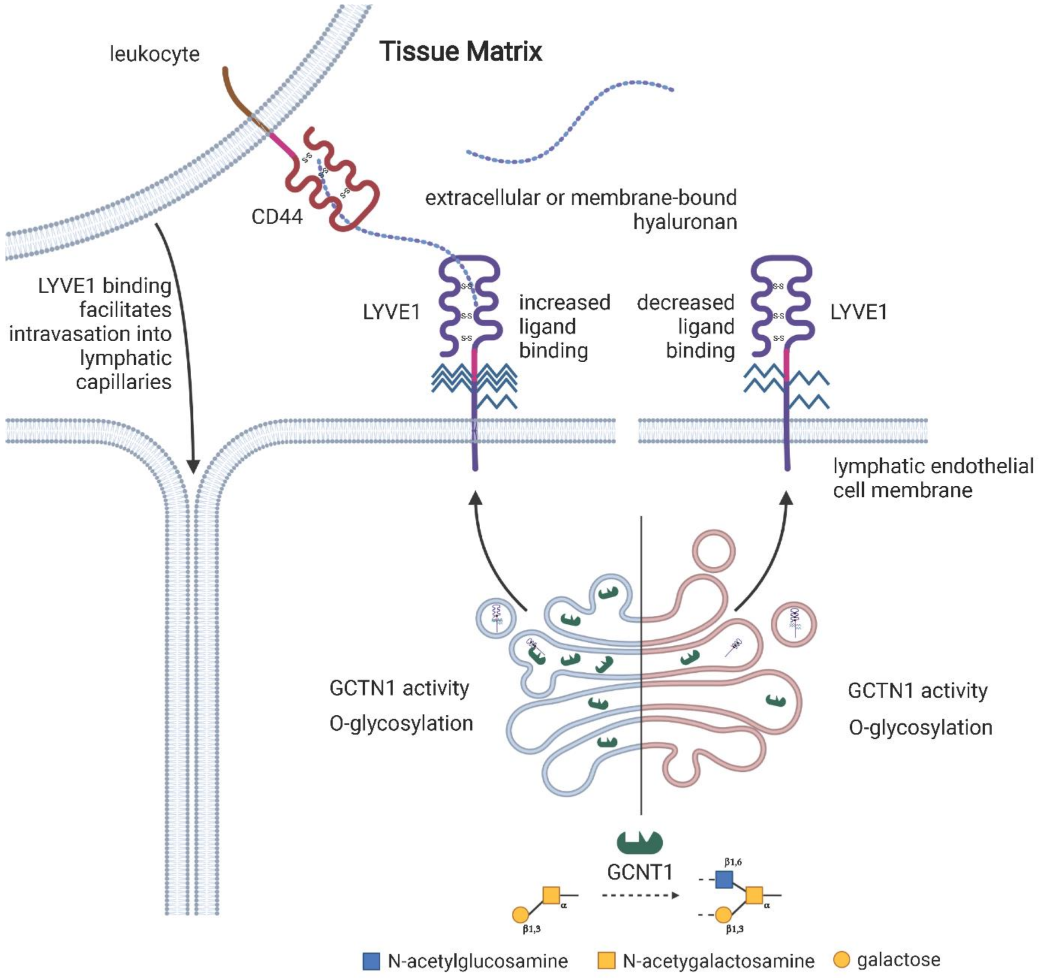

The low-frequency *GCNT1* missense rs147866228 (MAF=0.0491, β=-0.563, σ=0.0746, $p=4.31x10^{-14}$) is associated with decreased levels of lymphatic vessel endothelial hyaluronan receptor 1 (LYVE1). LYVE1

is a CD44 homolog found primarily on the surface of lymphatic endothelial cells. It binds to both hyaluronic acid and CD44, and is able to transport leukocytes into the lymphatic lumen[19,20]. It also has a homeostatic role in maintaining arterial tone[21]. In keeping with this role, PheWAS shows that this variant is a known monocyte-decreasing signal[22-24]. The signal co-localises with *GCNT1* eQTL in the oesophagus mucosa, *RFK* eQTL in thyroid tissue, and *PRUNE2* eQTL in whole blood. GCNT1 is one of three glycosyltransferases that construct the core 2 O-glycan branch, a type of O-glycan structure that is commonly found on O-glycoproteins[25]. LYVE-1's extracellular stalk domain is heavily O-glycosylated[26], and de-glycosylation greatly increases its HA-binding activity[27]. This signal could therefore represent molecular evidence for GCTN1-mediated regulation of LYVE1 HA binding and its subsequent activity in lymphatic and arterial vessels. rs147866228 has increased in frequency 9.82-fold compared to gnomAD exomes non-Finnish Europeans, and we do not detect any notable signal in the genomic regions of GCNT1 partners GCNT3 and GCNT4. LYVE1 is an emerging immunomodulating target for disorders of chronic inflammation[19], and GCNT1-linked O-glycosylation may represent a novel gateway to intervention.

TGFBR3 *trans KNG1* missense

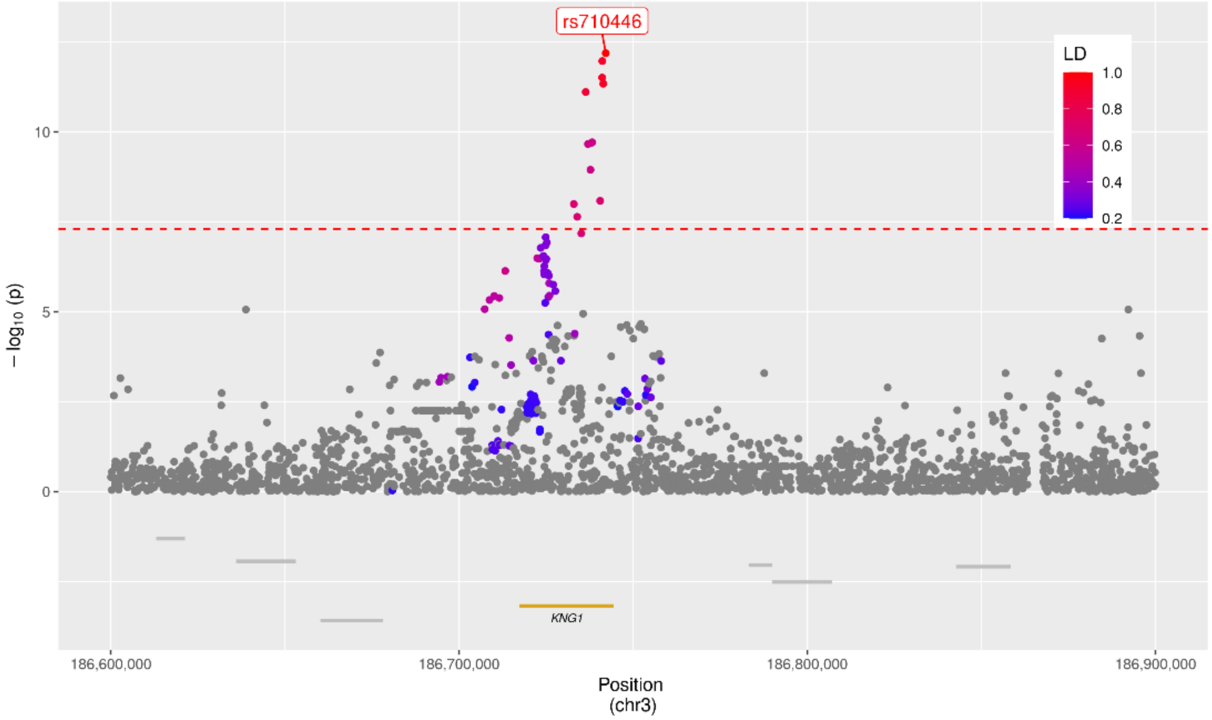

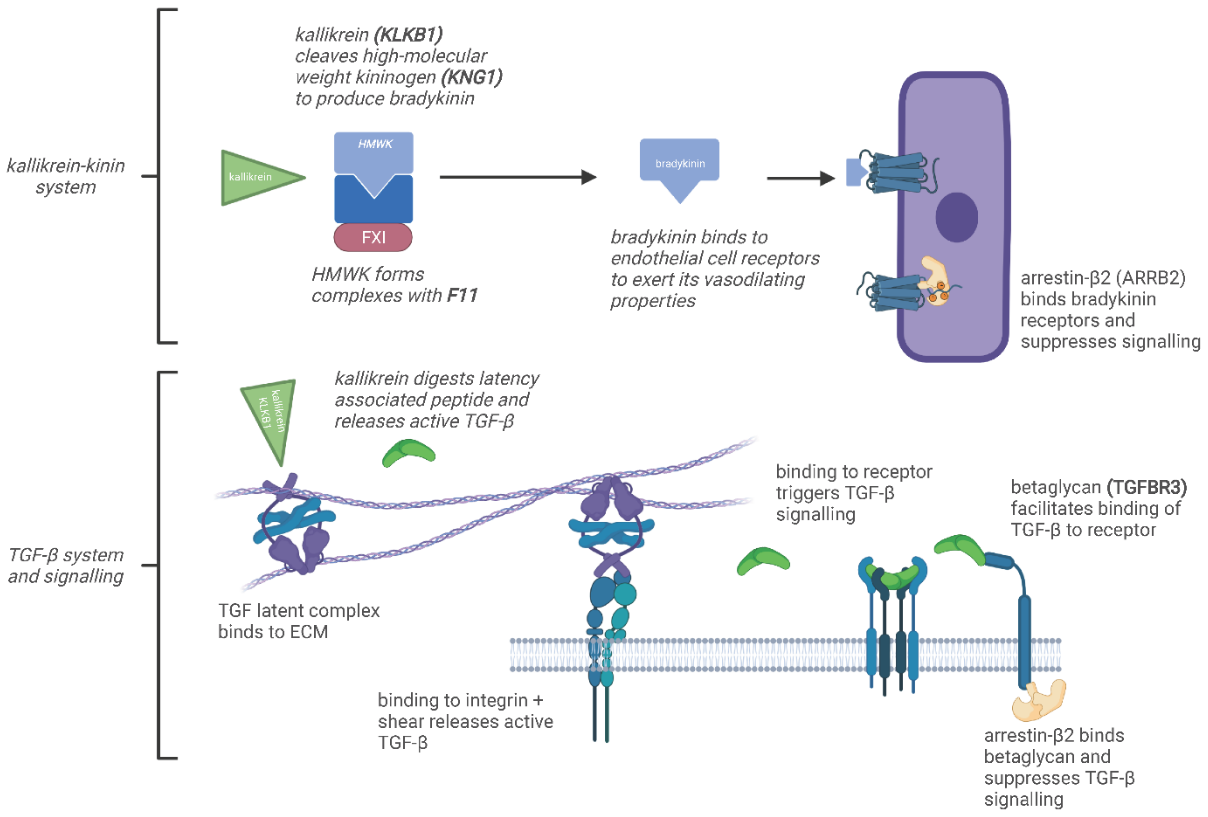

The common *KNG1* missense rs710446 (MAF=0.4972, β=0.1941, σ=0.027, $p=6.54\times10^{-13}$) is associated with an increase in serum levels of betaglycan (TGFBR3). The same SNV is associated with increased levels of F11, KLKB1, KNG1, MAN1B1, STX7, as well as increased kallikrein, bradykinin and renin activity. Other phenotypes implicated at this signal by PheWAS include activated partial thromboplastin time, venous thromboembolism and thrombosis.

TGFBR3 is a co-receptor for the TGF-β superfamily, presenting ligands to receptors. It broadly influences the activities and interactions of a number of TGF superfamily members, thereby impacting diverse cellular processes[28].

KNG1 encodes kininogen, the precursor protein of the kallikrein-kinin system (KKS), a pathway related to the coagulation-inducing contact activation system (CAS), which culminates in the release of the vasodilator bradykinin(BK). Consistent with this role, MR showed this variant to have a causal systolic and diastolic blood pressure-increasing effect. Interestingly, the same variant was causally protective against Stroke, but increased risk of general and Hip/Knee osteoarthritis. KLKB1 (Kallikrein) also belongs to the KKS, and F11 is an indirect modulator as well as a HMWK ligand[29], explaining previous pQTL signals for these two proteins at the *KNG1* locus.

The KKS interacts with the TGF-β pathway, with plasma KLKB1 cleaving TGF-β1 latency-associated peptide [30]. Kininogen-deficient mice display increased levels of TGF-β1, a pro-fibrotic factor[31] which may displays anti-inflammation effects in ischaemic stroke[32]. Kallikrein inhibition similarly protected against thromboinflammation and renal damage in mice and rats[33-35], and treatment of human acute ischaemic stroke with kallikrein has been approved in some countries[36]. BK receptor blockade increased TGF-β1 and BK infusion reduces fibrosis and TGF-β1[31].

This signal suggests that betaglycan abundance and/or activity is impacted as part of the TGF-B modulating effect of the KKS.

TIE1 *trans RHBDF1* missense

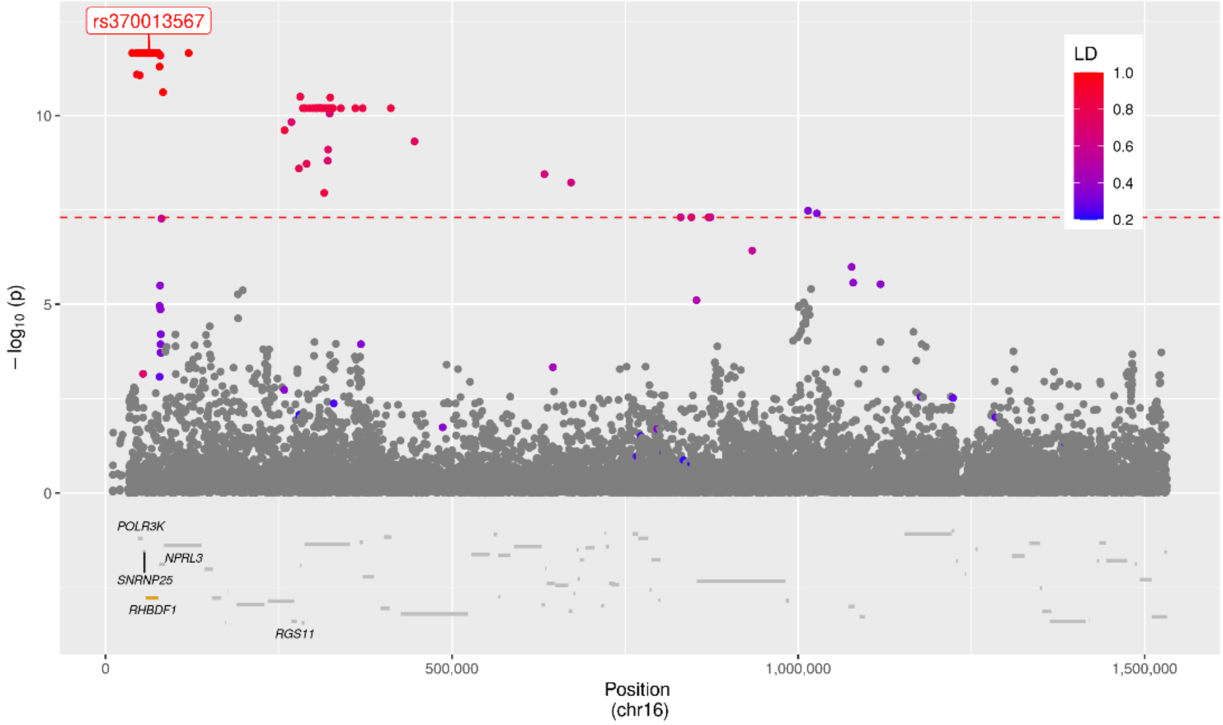

The low-frequency *RHBDF1* missense rs370013567 (MAF=0.0131, β=-1.1453, σ=0.1631, p=2.20x10^{-12}) is associated with decreased levels of Tyrosine Kinase With Immunoglobulin Like And EGF Like Domains 1 (TIE1). The association is located at the alpha globin locus at the start of chromosome 16, and involves a number of SNVs associated at identical p-value in a plateau, making causal gene identification difficult. The signal co-localises with eQTL for a number of genes in multiple tissues including *SNRNP25, POLR3K, NPRL3,* and *RGS11,* but not *RHBDF1*. Interestingly, no association signal is present for TIE2 at this locus (lowest association P-value 0.39), suggesting that this particular interaction is specific to TIE1. TIE1 is an endothelial cell specific orphan receptor that has been shown to modulate TIE2/angiopoietin signalling, which controls angiogenesis and vessel maturation[37-39]. Deletion of either receptor is embryonic lethal[40].

CCL5 *trans NFKB2*

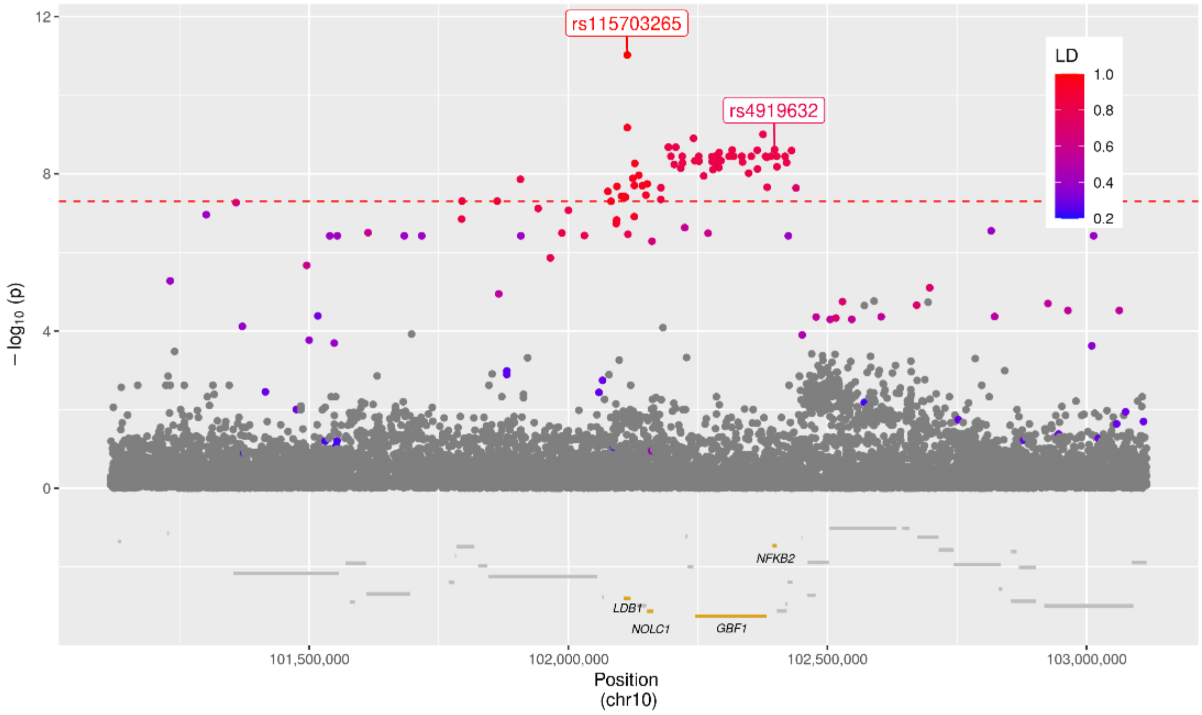

The intronic *LDB1* variant rs115703265 (MAF=0.0474, β=0.6053, σ=0.0888, $p=9.57x10^{-12}$) is associated with increased levels of the inflammatory chemokine CCL5 (Chemokine Ligand 5). It is a late-stage chemotactic attractant for many leukocyte subtypes, involved in maintaining inflammation several days after bacterial or viral infection. It is expressed in many cell types, including T-cells, monocytes, epithelial cells and fibroblasts. The signal colocalizes with a *NOLC1* eQTL in the cerebellum. Moreover, variants in high LD with the lead SNV are eQTL for 13 further genes in the region. Two genes in the region, *GBF1* and *NFKB2,* are involved in the immune response, despite not having eQTL overlapping with our signal. The guanine nucleotide exchange factor GBF1 is canonically involved in intracellular transport between the Golgi apparatus and Endoplasmic reticulum. However, it is also an important modulator of the RNA replication and virion assembly for many viruses[41]. Secondly, rs115703265 is in strong LD (r^2=0.83) with rs4919632, an intronic *NFKB2* variant annotated by ClinVar as involved in common variable immunodeficiency 10. This disorder, caused by high-impact variants in *NFKB2*, is characterized by childhood-onset recurrent infections[42-48]. Upregulation of GBF1 or impairment of NFKB2 could both lead to longstanding and increased infection and viral load, thereby soliciting an enhanced chemotactic response by CCL5. However, the links are speculative as no link via eQTL or other co-expression can be established.

TIMP1 *trans TENT5C*

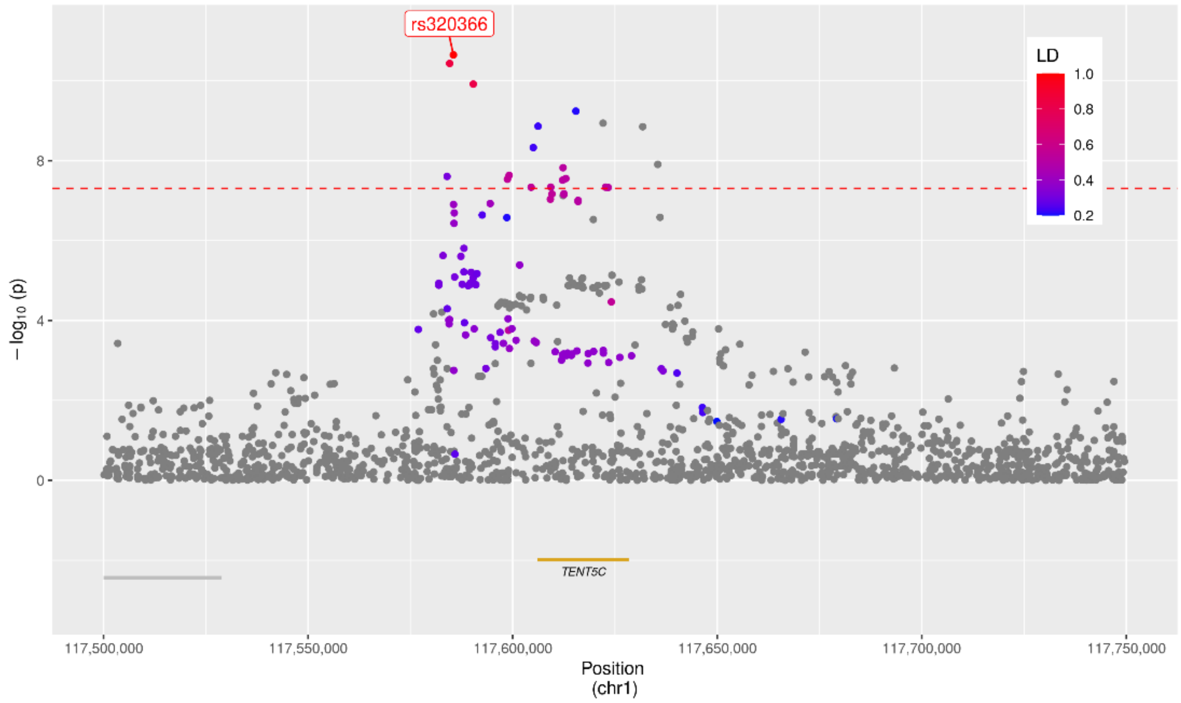

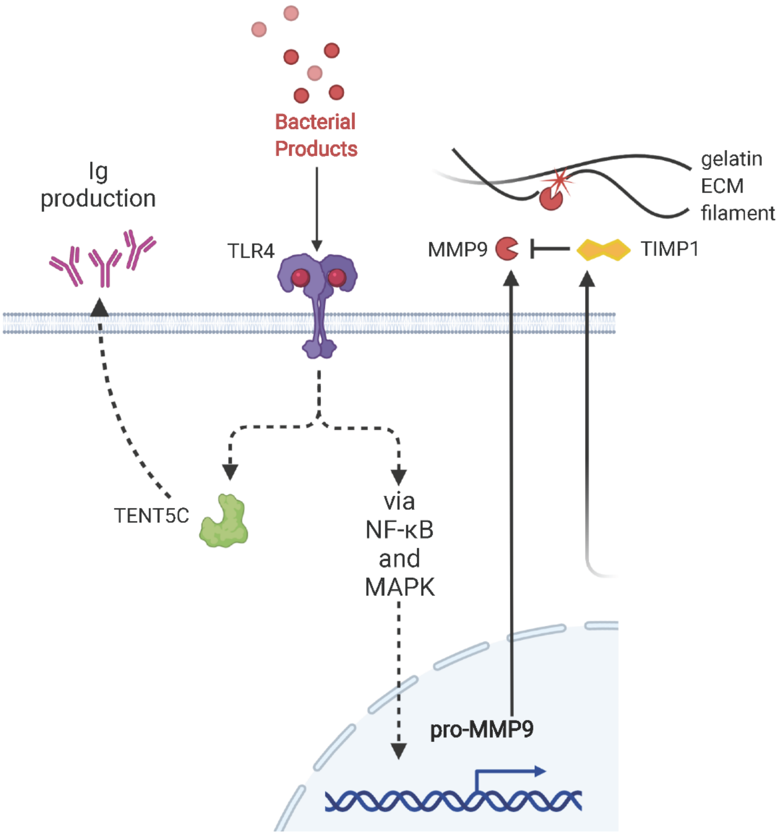

TIMP metallopeptidase 1 (TIMP1) is a polyvalent tissue inhibitor of metalloproteinases that targets 14 of the 24 known MMPs[49]. The balance between MMPs and TIMPs also regulates other acute response processes. The intergenic common rs320366 was associated with a decrease of TIMP1 (MAF=0.3501, β=-0.192, σ=0.0287, p=2.26x10^{-11}). This signal colocalized with *TENT5C* eQTL in multiple tissues.

Since nearly all mRNA decay pathways begin with the removal of poly(A) tails, polyadenylation at the 3' end of mRNA enhances its stability. Polyadenylation is mostly mediated through canonical, nucleic canonical poly(A) polymerases. However, the poly(A) tail can be expanded in the cytoplasm by non-canonical poly(A) polymerases (ncPAPs). TENT5C is a member of this family, which preferentially targets ER-targeted protein mRNA. It is uniquely and highly expressed in embryonic stem cells and its deletion is embryonic lethal[50]. It was also shown to stimulate the transition of naïve B lymphocytes into antibody secreting cells and the production of immunoglobulins[51]. TENT5C expression is upregulated by stimulation of TLR1,2,4,6,9. Independently of this B-cell specific polyadenylation activity, TENT5C inhibits the tumor growth factor Plk4, which is concordant with the high observed frequency of null mutations in multiple myeloma patients[52].

TIMP1 is both a proteolytic agent targeting metalloproteinases, and a signaling molecule, known for its profibrotic role and ambiguous effect in cancer[53]. An important ligand of TIMP1 is pro-MMP9, whose expression is stimulated in response to TLR4 activation through the MAPK/NF-kB pathways[54,55]. NF-κB is a family of inducible transcription factors that stimulates immune responses. It is normally sequestered in the cytoplasm, but moves to the nucleus to exert transcriptional functions in response to stress and immune stimuli[56].

This signal may therefore reflect either direct targeting of TIMP1 mRNA by TENT5C as part of its polyadenylating activity, or co-activation of TENT5C expression and NF-kB-mediated MMP-9 production, which is specifically inhibited by TIMP1. TENT5C levels are not available in our cohorts, and neither TIMP1 nor MMP9 display a signal in the TLR gene regions. There is a TIMP1 signal in the MMP9 gene region driven by a rare variant at $p=6.81\times10^{-6}$.

TGFBI *trans ORM1*

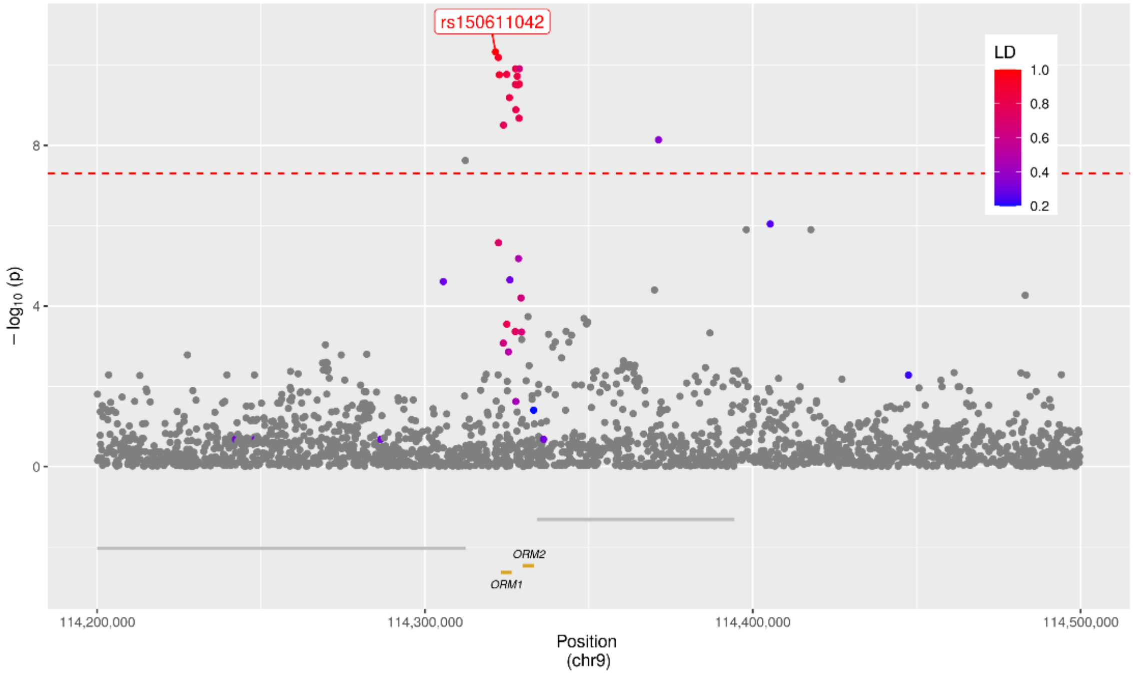

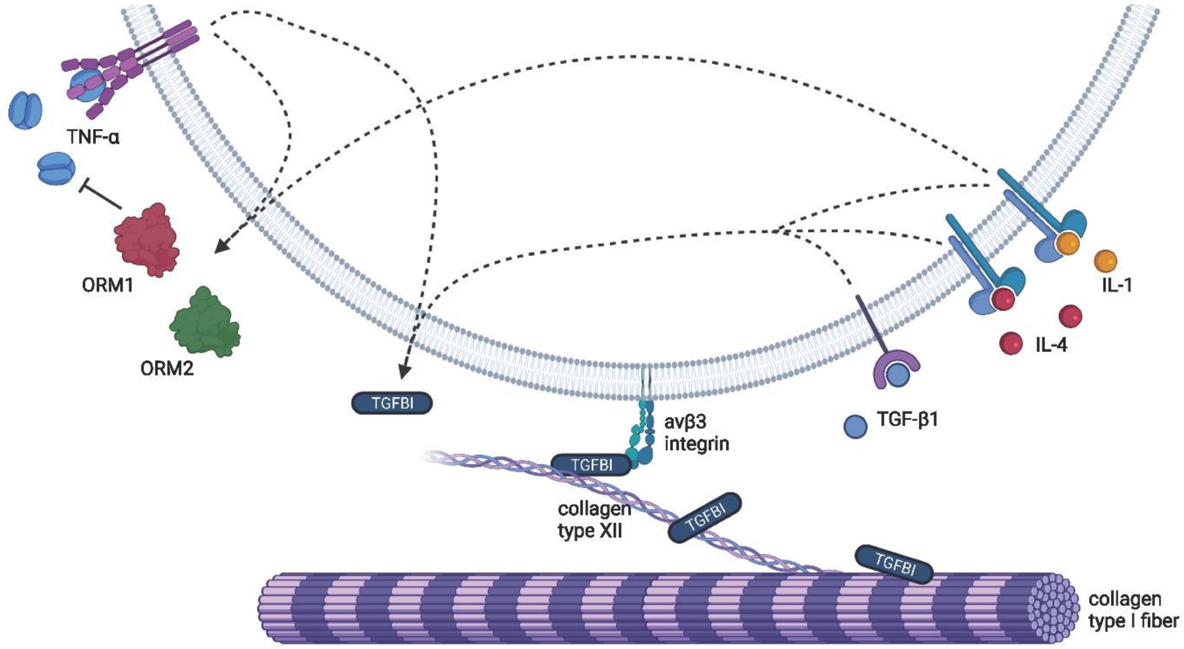

The common regulatory region variant rs150611042 (MAF=0.068, β=-0.362, σ=0.055, $p=4.69\times10^{-11}$) is associated with decreased levels of transforming growth factor, beta induced (TGFBI). The signal colocalises with eQTL for *ORM1* and *ORM2* in multiple tissues, as well as with an HDL-increasing and cholesterol, triglyceride and LDL-lowering signal. Variants tagging rs150611042 were associated with decreased thrombin generation potential, increased testosterone, increased F8, SPINK2 and VWF, decreased TFPI and F10. The orosomucoid (ORM) genes are members of the lipocalin family that encode acute-phase proteins upregulated by glucocorticoids, TNF-α, IL-1, IL-8, IL-11, although their biological function remains largely unknown. They are primarily produced by hepatocytes, but can also be synthesized by leukocytes and endothelial cells. ORMs are inhibited by estrogen[57] and are able to bind progesterone[58]. They are also known for their ability to bind more than 300 drugs, impacting their bioavailability, volume of distribution, metabolism, and therapeutic effect[59]. ORMs are believed to be bidirectional modulators of inflammation in acute phase response[60].

TGFBI is a ubiquitously expressed TGF-β-induced extracellular matrix protein that binds type-I, II, IV and VI collagens, fibronectin, periostin, laminin, biglycan and decorin[61]. Integrins, which are important vectors of cell adhesion, act as surface receptors for TGFBI which is able to recruit them to support cell adhesion. It plays a critical role in corneal health, and *TGFBI* point mutations are responsible for monogenic corneal dystrophies[62]. Lipocalins, in particular TLC/LCN1, are important for eye health, and ORM1 has been associated with dry eye syndrome. Importantly, TGFBI expression is also induced by IL-4, IL-1β, and TNF-α[63], and can induce signalling activation and up-regulation of NF-κB to stimulate immune responses[56]. This *trans* signal may reflect immune-mediated co-activation of TGFBI and ORM in response to TNF-α and IL-1 signalling. Interestingly, ORM1 has previously been shown to interact with SERPINE1[64], which is often seen upregulated along with TGFBI, with which it shares a TGF-β-driven activation mechanism.

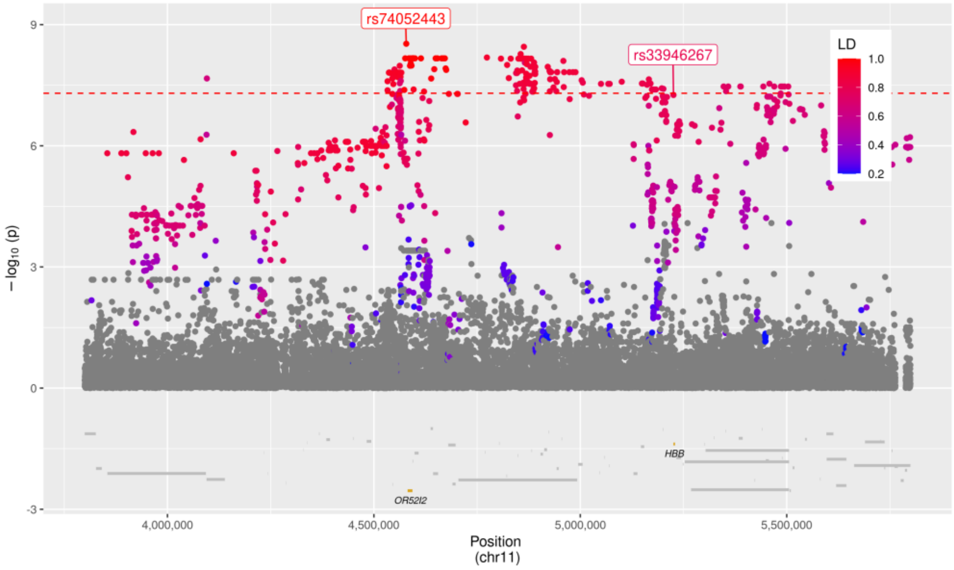

VCAM1 *trans HBB*

A low-frequency regulatory region variant rs74052443 (MAF=0.041, β=-0.566, σ=0.0954, $p=2.97\times10^{-9}$) showed association with decreased level of Vascular cell adhesion protein 1(VCAM1). rs74052443 overlaps *OR52I2* gene's regulatory region. OR5212 encodes olfactory receptor 52I2 where no clear biological link can be established with a decreased level of VCAM1 protein. However, rs33946267 (MAF=0.0461, β=0.494, σ=0.0909, $p=5.52\times10^{-8}$) variant with a stop-gained consequence on the *HBB* gene is in LD with rs74052443.

HBB gene encodes hemoglobin subunit β. one α1, one α2, and two β subunits together form the oxygen carrying hemoglobin. Mutations in the *HBB* gene is known to cause sickle cell disease (SCD)[65]. Sickle cell disease is characterised by the rigid sickle-shaped red blood cells (RBC) due to the abnormal β subunit of hemoglobin[66,67]. Severity of SCD is highly variable across individuals[68,69], but individuals with SCD are generally under chronic inflammatory state through various mechanisms [70]. The two main mechanisms are hemolytic anemia and vaso-occlusion crisis. Sickle-shaped RBCs in comparison to normal RBCs are structurally rigid, therefore have a much shorter lifespan as they tend to breakdown prematurely and release heme and DAMP (damage-associated molecular patterns). In addition, sickle-shaped RBCs may cause blood vessel blockage, causing vaso-occlusion crisis and leading to cell necrosis in oxygen-deprived tissues and organs. Release of cellular molecules from cell necrosis are detected by leukocytes which initiates inflammatory response by leukocytes releasing pro-inflammatory cytokines including tumour necrosis factor alpha (TNFα). TNFα binds to TNF receptor 1 (TNFR1) proteins, which induces expression of various genes downstream, one of which is the *VCAM1* gene[71]. *VCAM1* gene is mainly expressed in endothelial cells and encodes a transmemebrane protein which binds to α4β1 integrins on leukocyte cell surface to initiate transmigration of leukocytes across tissues in order to traffick leukocytes into sites of inflammation[71].

HBB gene appears to have an indirect link to VCAM1 via TNFα and TNFR1 through inflammatory events caused by SCD. Indeed, severe sickle cell anemia has been associated with increased TNFR1 and VCAM1 levels in the past [72]. However, there are 349 variants in LD>0.8, which makes it difficult to determine whether the *HBB* stop-gained rs33946267 variant is truly the causative variant for the increase in VCAM1 abundance. In addition, no significant *HBB* eQTL colocalisation nor VCAM1 pQTL signal in *TNFR1* gene region were detected in our data.

Novel *trans* signals in main text involved in inflammatory and immune response

We outline the involvement of the novel *trans* signals mentioned in the main text to inflammatory and immune response (Supplementary Figure 5).

Platelet activation

We find genetic evidence that the counterbalancing actions of ADAM17 and TIMP3 controls shedding of platelet surface receptors such as GP1BA critical in platelet activation. Platelet activation is one of the functions effected by the AXL/GAS6 axis, for which we document a novel *trans* association, and which is central in injury and inflammation. Its activation has been linked to further related mechanisms, such as apoptotic cell clearance[73], leukocyte migration, endothelial cell activation, adhesion, proliferation and chemokine expression[74,75].

Acute-phase response

We describe two *trans* associations involving members of the lipocalin gene family, *ORM1/2* and *LCN2*. Lipocalins are a group of extracellular transport proteins that bind small molecules and are involved in the metabolism of drugs, vitamins, metabolites and hormones. Both ORM1 and LCN2 are acute-phase response proteins upregulated in wounded tissue. The former is chiefly expressed in the liver and has a potent anti-inflammatory action exerted through inhibition of chemotaxis, superoxide production, lymphocyte proliferation, platelet aggregation, and antagonization of bradykinin[60,76]. We find *ORM1* regulatory variants influencing levels of transforming growth factor, beta induced (TGFBI), which may reflect co-activation in response to TNF-α and IL-1 signalling. The modulation of LCN2, a pro-inflammatory bactericidal constituent of neutrophil secondary granules, by complement factor H variants provides genetic evidence linking lipocalin function to the complement cascade and downstream processes such as platelet activation[77].

Vasodilation

We find a *trans*-acting *KNG1* missense variant increasing betaglycan (TGFBR3) levels. KNG1 encodes kininogen, the main precursor of the kallikirein-kinin system (KKS),

which produces the vasodilator bradykinin and leads to increased permeability of blood vessels close to the site of inflammation. Betaglycan is a broad-acting co-receptor in the TGF-βpathway, which stimulates ECM growth and acts as a potent regulator of inflammation and immune response[78,79].

Immune cell trafficking

LYVE1 traffics dendritic cells and macrophages through lymphatic vessels, processes that are key both for immune priming and the resolution of inflammation[80]. Genetic evidence in *trans* linking it to the O-glycosyltransferase GCTN1 may hint at a key regulatory mechanism of LYVE1 function.

Supplementary Figures

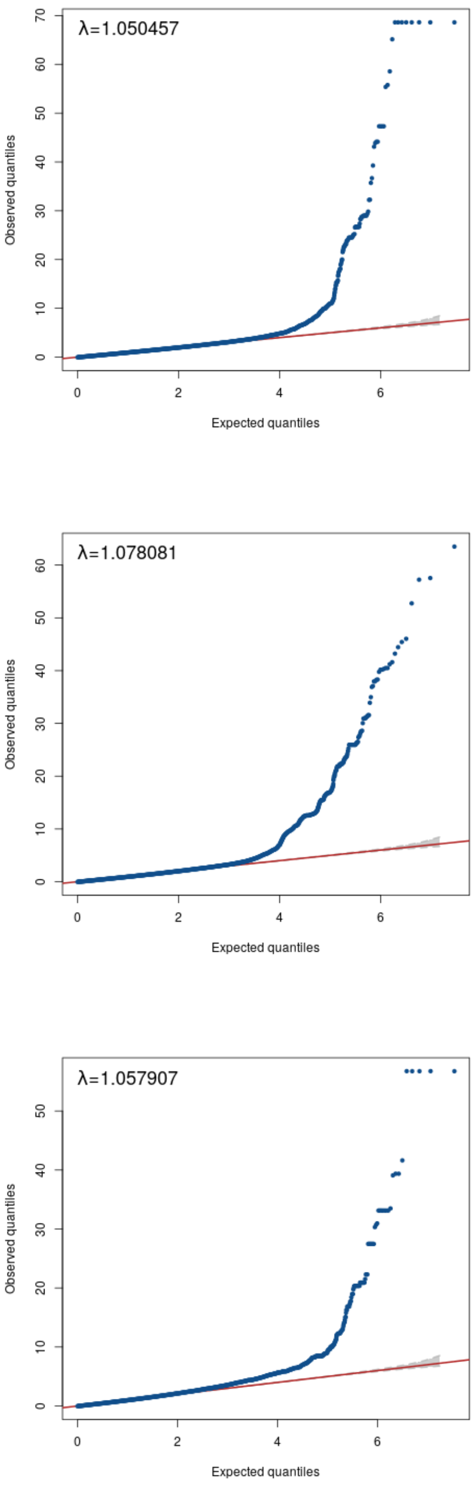

Supplementary Figure 1. QQ-plot of three proteins exceeding genomic inflation factor. QQ-plot of Pomak cohort's CD46 protein (top), C2 (middle), and MANOLIS QPCT (bottom) is shown. Genomic inflation factor of each protein association is displayed within the graph on the top-left region.

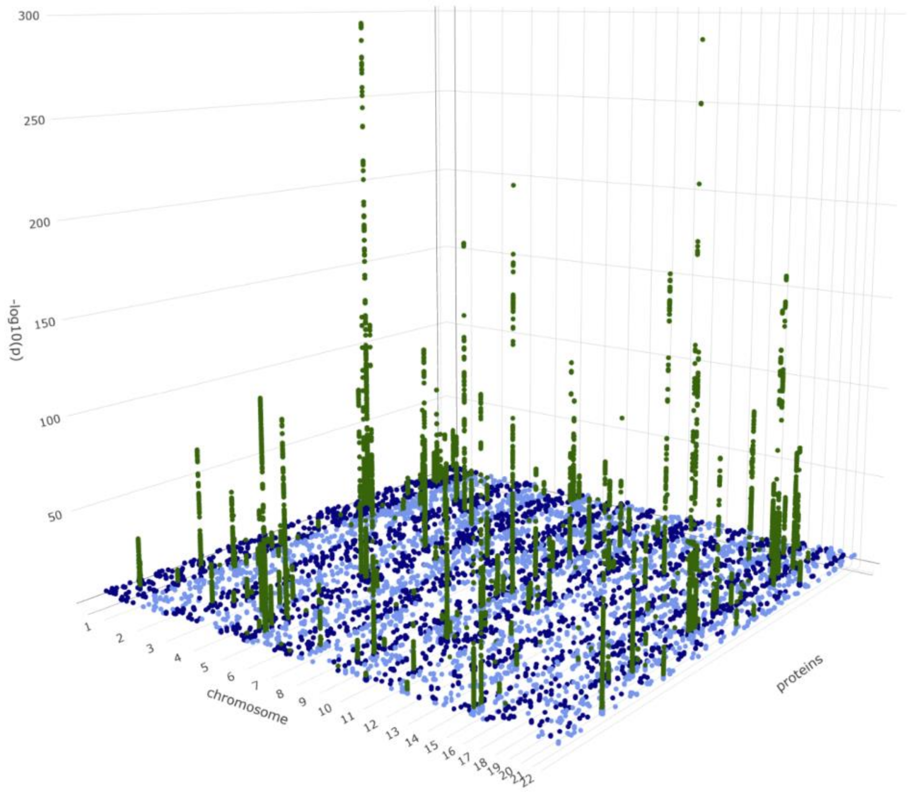

Supplementary Figure 2. Genome-wide pQTL signals across all tested cardiometabolic proteins. Only $p<1x10^{-5}$ are shown in the plot with $p<5x10^{-8}$ signals highlighted in dark green.

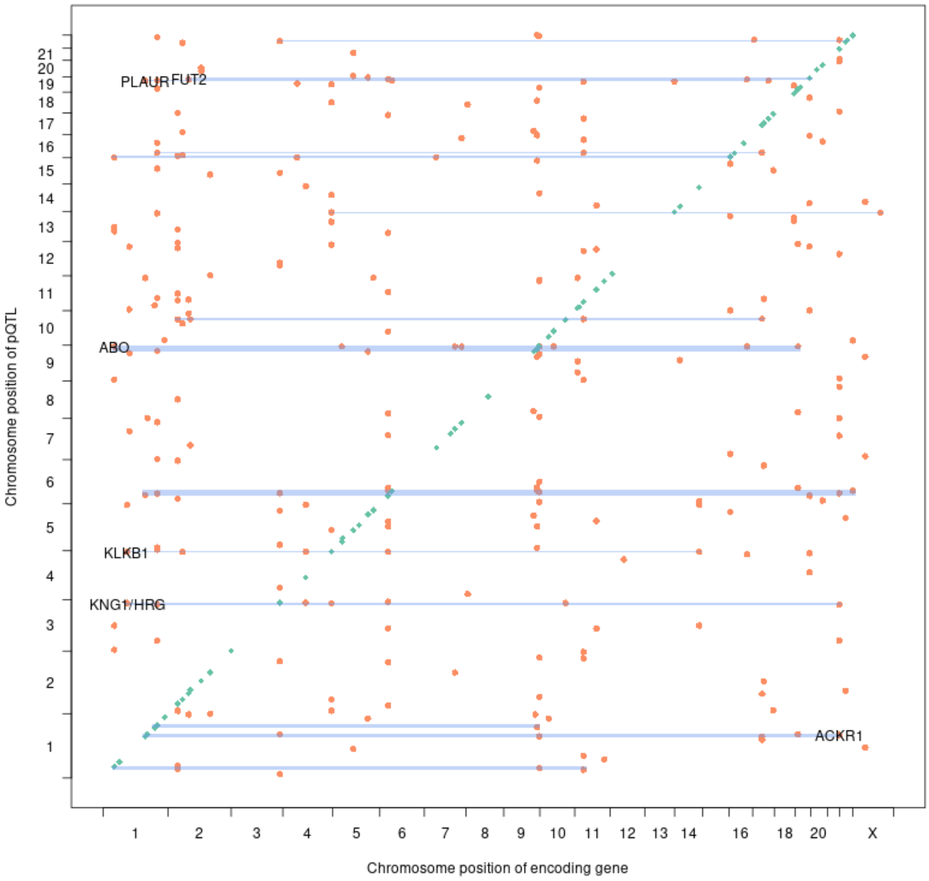

Supplementary Figure 3. Scatterplot of pQTL signals with highlighted multi-signal loci. X-axis depicts the position of gene encoding the protein trait, while the y-axis depicts the pQTL signal loci for the protein trait. Orange circles and teal diamonds represents *trans* and *cis*-pQTLs, respectively. Loci with 4 or more overlapping pQTL signals are highlighted with transparent blue band, and bands overlapping well-known pleiotropic genes are labelled with the pleiotropic gene name.

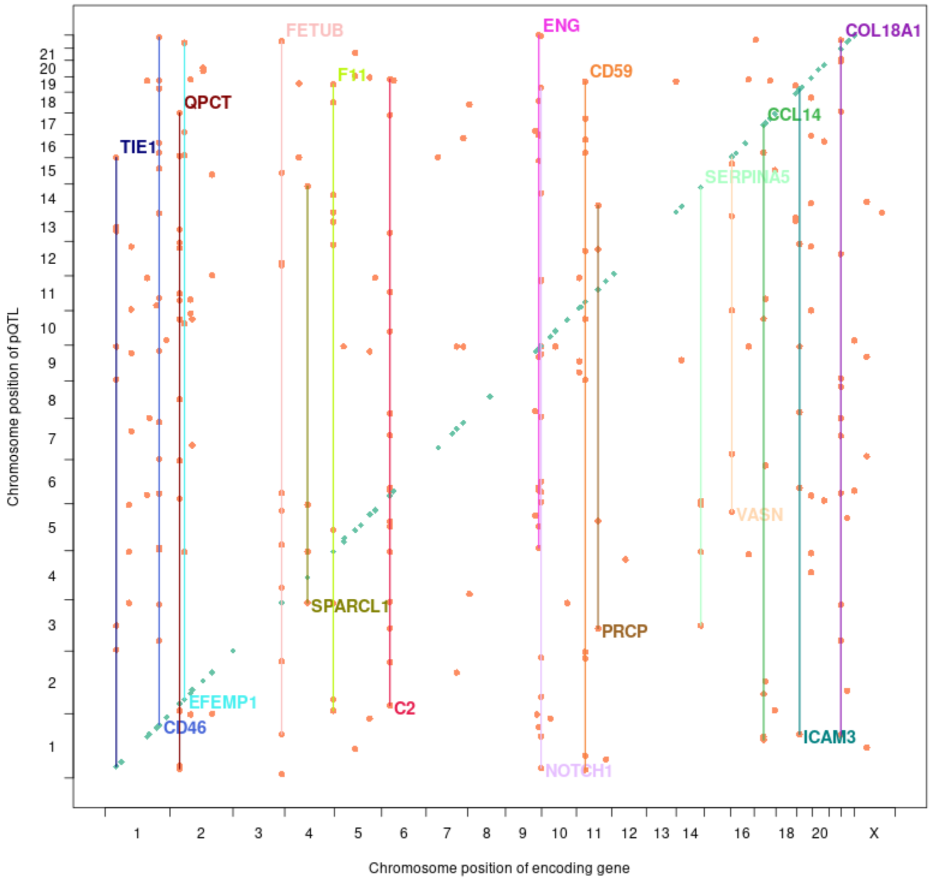

Supplementary Figure 4. Scatterplot of pQTL signals and proteins with multiple pQTL signals highlighted. Axis and datapoint representations are identical with Supplementary Figure 3. pQTL datapoints from each protein with 4 or more signals are connected together with a distinctly coloured line and labelled with the protein name.

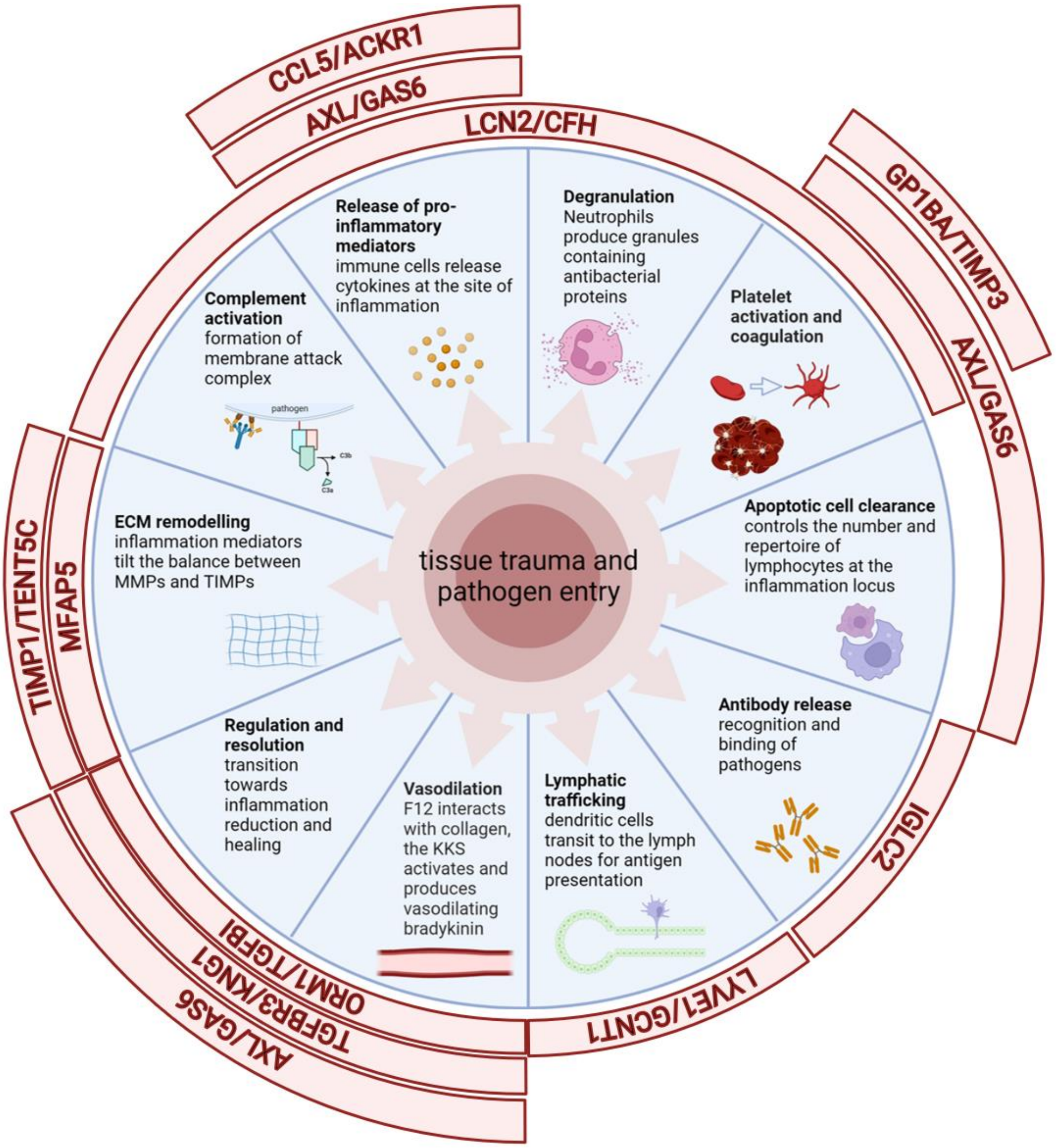

Supplementary Figure 5. Function and context of IGLC2 and MFAP5, for which *cis* associations were described, as well as for six *trans* protein pairs (Table 1), in acute inflammation and immune response cascades. Complement activation schematic credit to Akiko Iwasaki and Ruslan Medzhitov. Created using BioRender.

References

1. Gudjonsson, A. *et al.* A genome-wide association study of serum proteins reveals shared loci with common diseases. *Nat Commun* **13**, 480 (2022).
2. Ge, T. *et al.* The Role of the Pentose Phosphate Pathway in Diabetes and Cancer. *Front Endocrinol (Lausanne)* **11**, 365 (2020).

3. Vaittinen, M. *et al.* MFAP5 is related to obesity-associated adipose tissue and extracellular matrix remodeling and inflammation. *Obesity (Silver Spring)* **23**, 1371-8 (2015).
4. Wu, Z. *et al.* MFAP5 promotes tumor progression and bone metastasis by regulating ERK/MMP signaling pathways in breast cancer. *Biochem Biophys Res Commun* **498**, 495-501 (2018).
5. Fingleton, B. Matrix metalloproteinases as regulators of inflammatory processes. *Biochim Biophys Acta Mol Cell Res* **1864**, 2036-2042 (2017).
6. Vaittinen, M., Kolehmainen, M., Schwab, U., Uusitupa, M. & Pulkkinen, L. Microfibrillar-associated protein 5 is linked with markers of obesity-related extracellular matrix remodeling and inflammation. *Nutr Diabetes* **1**, e15 (2011).
7. Barbier, M. *et al.* MFAP5 loss-of-function mutations underscore the involvement of matrix alteration in the pathogenesis of familial thoracic aortic aneurysms and dissections. *Am J Hum Genet* **95**, 736-43 (2014).
8. Roychowdhury, T. *et al.* Regulatory variants in TCF7L2 are associated with thoracic aortic aneurysm. *Am J Hum Genet* **108**, 1578-1589 (2021).
9. Novitzky-Basso, I. & Rot, A. Duffy antigen receptor for chemokines and its involvement in patterning and control of inflammatory chemokines. *Front Immunol* **3**, 266 (2012).
10. Luo, H., Chaudhuri, A., Zbrzezna, V., He, Y. & Pogo, A.O. Deletion of the murine Duffy gene (Dfy) reveals that the Duffy receptor is functionally redundant. *Mol Cell Biol* **20**, 3097-101 (2000).
11. Horuk, R. The Duffy Antigen Receptor for Chemokines DARC/ACKR1. *Front Immunol* **6**, 279 (2015).
12. Abella, V. *et al.* The potential of lipocalin-2/NGAL as biomarker for inflammatory and metabolic diseases. *Biomarkers* **20**, 565-71 (2015).
13. Chakraborty, S., Kaur, S., Guha, S. & Batra, S.K. The multifaceted roles of neutrophil gelatinase associated lipocalin (NGAL) in inflammation and cancer. *Biochim Biophys Acta* **1826**, 129-69 (2012).
14. Guo, B.X. *et al.* Lipocalin 2 regulates intestine bacterial survival by interplaying with siderophore in a weaned piglet model of Escherichia coli infection. *Oncotarget* **8**, 65386-65396 (2017).
15. Flo, T.H. *et al.* Lipocalin 2 mediates an innate immune response to bacterial infection by sequestrating iron. *Nature* **432**, 917-21 (2004).
16. Nelson, A.L., Ratner, A.J., Barasch, J. & Weiser, J.N. Interleukin-8 secretion in response to aferric enterobactin is potentiated by siderocalin. *Infect Immun* **75**, 3160-8 (2007).
17. Bachman, M.A., Miller, V.L. & Weiser, J.N. Mucosal lipocalin 2 has pro-inflammatory and iron-sequestering effects in response to bacterial enterobactin. *PLoS Pathog* **5**, e1000622 (2009).
18. Gilly, A. *et al.* Gene-based whole genome sequencing meta-analysis of 250 circulating proteins in three isolated European populations. *Mol Metab* **61**, 101509 (2022).
19. Rinaldi, E. & Baggi, F. LYVE-1 is 'on stage' now: an emerging player in dendritic cell docking to lymphatic endothelial cells. *Cell Mol Immunol* **15**, 663-665 (2018).
20. Johnson, L.A. & Jackson, D.G. Hyaluronan and Its Receptors: Key Mediators of Immune Cell Entry and Trafficking in the Lymphatic System. *Cells* **10**(2021).
21. Lim, H.Y. *et al.* Hyaluronan Receptor LYVE-1-Expressing Macrophages Maintain Arterial Tone through Hyaluronan-Mediated Regulation of Smooth Muscle Cell Collagen. *Immunity* **49**, 326-341 e7 (2018).
22. Chen, M.H. *et al.* Trans-ethnic and Ancestry-Specific Blood-Cell Genetics in 746,667 Individuals from 5 Global Populations. *Cell* **182**, 1198-1213 e14 (2020).
23. Barton, A.R., Sherman, M.A., Mukamel, R.E. & Loh, P.R. Whole-exome imputation within UK Biobank powers rare coding variant association and fine-mapping analyses. *Nat Genet* **53**, 1260-1269 (2021).
24. Vuckovic, D. *et al.* The Polygenic and Monogenic Basis of Blood Traits and Diseases. *Cell* **182**, 1214-1231 e11 (2020).
25. Marth, J.D. & Grewal, P.K. Mammalian glycosylation in immunity. *Nat Rev Immunol* **8**, 874-87 (2008).

26. Jackson, D.G. The lymphatic endothelial hyaluronan receptor LYVE-1. *Glycoforum* **8**(2004).
27. Nightingale, T.D., Frayne, M.E., Clasper, S., Banerji, S. & Jackson, D.G. A mechanism of sialylation functionally silences the hyaluronan receptor LYVE-1 in lymphatic endothelium. *J Biol Chem* **284**, 3935-45 (2009).
28. Bilandzic, M. & Stenvers, K.L. Betaglycan: a multifunctional accessory. *Mol Cell Endocrinol* **339**, 180-9 (2011).
29. Bekassy, Z., Lopatko Fagerstrom, I., Bader, M. & Karpman, D. Crosstalk between the renin-angiotensin, complement and kallikrein-kinin systems in inflammation. *Nat Rev Immunol* **22**, 411-428 (2022).
30. Robertson, I.B. & Rifkin, D.B. Regulation of the Bioavailability of TGF-beta and TGF-beta-Related Proteins. *Cold Spring Harb Perspect Biol* **8**(2016).
31. Acuna, M.J. *et al.* Blockade of Bradykinin receptors worsens the dystrophic phenotype of mdx mice: differential effects for B1 and B2 receptors. *J Cell Commun Signal* **12**, 589-601 (2018).
32. Langhauser, F. *et al.* Kininogen deficiency protects from ischemic neurodegeneration in mice by reducing thrombosis, blood-brain barrier damage, and inflammation. *Blood* **120**, 4082-92 (2012).
33. Gob, E. *et al.* Blocking of plasma kallikrein ameliorates stroke by reducing thromboinflammation. *Ann Neurol* **77**, 784-803 (2015).
34. Xia, C.F. *et al.* Kallikrein protects against ischemic stroke by inhibiting apoptosis and inflammation and promoting angiogenesis and neurogenesis. *Hum Gene Ther* **17**, 206-19 (2006).
35. Chao, J., Bledsoe, G., Yin, H. & Chao, L. The tissue kallikrein-kinin system protects against cardiovascular and renal diseases and ischemic stroke independently of blood pressure reduction. *Biol Chem* **387**, 665-75 (2006).
36. Alexander-Curtis, M. *et al.* Human tissue kallikrein in the treatment of acute ischemic stroke. *Ther Adv Neurol Disord* **12**, 1756286418821918 (2019).
37. Mueller, S.B. & Kontos, C.D. Tie1: an orphan receptor provides context for angiopoietin-2/Tie2 signaling. *J Clin Invest* **126**, 3188-91 (2016).
38. Korhonen, E.A. *et al.* Tie1 controls angiopoietin function in vascular remodeling and inflammation. *J Clin Invest* **126**, 3495-510 (2016).
39. Leppanen, V.M., Saharinen, P. & Alitalo, K. Structural basis of Tie2 activation and Tie2/Tie1 heterodimerization. *Proc Natl Acad Sci U S A* **114**, 4376-4381 (2017).
40. Thomas, K.A. Angiogenesis. in *Encyclopedia of Cell Biology* (eds. Bradshaw, R.A. & Stahl, P.D.) 102-116 (Academic Press, Waltham, 2016).
41. Martinez, J.L. & Arias, C.F. Role of the Guanine Nucleotide Exchange Factor GBF1 in the Replication of RNA Viruses. *Viruses* **12**(2020).
42. Aird, A. *et al.* Novel Heterozygous Mutation in NFKB2 Is Associated With Early Onset CVID and a Functional Defect in NK Cells Complicated by Disseminated CMV Infection and Severe Nephrotic Syndrome. *Front Pediatr* **7**, 303 (2019).
43. Brue, T. *et al.* Mutations in NFKB2 and potential genetic heterogeneity in patients with DAVID syndrome, having variable endocrine and immune deficiencies. *BMC Med Genet* **15**, 139 (2014).
44. Chen, K. *et al.* Germline mutations in NFKB2 implicate the noncanonical NF-kappaB pathway in the pathogenesis of common variable immunodeficiency. *Am J Hum Genet* **93**, 812-24 (2013).
45. Hatfield, D. Oligonucleotide-ribosome-AA-sRNA interactions. *Cold Spring Harb Symp Quant Biol* **31**, 619-22 (1966).
46. Liu, Y. *et al.* Novel NFKB2 mutation in early-onset CVID. *J Clin Immunol* **34**, 686-90 (2014).
47. Quentien, M.H. *et al.* Deficit in anterior pituitary function and variable immune deficiency (DAVID) in children presenting with adrenocorticotropin deficiency and severe infections. *J Clin Endocrinol Metab* **97**, E121-8 (2012).
48. Tucker, E. *et al.* A novel mutation in the Nfkb2 gene generates an NF-kappa B2 "super repressor". *J Immunol* **179**, 7514-22 (2007).

49. Knight, B.E. *et al.* TIMP-1 Attenuates the Development of Inflammatory Pain Through MMP-Dependent and Receptor-Mediated Cell Signaling Mechanisms. *Front Mol Neurosci* **12**, 220 (2019).
50. Hu, J.L. *et al.* FAM46B is a prokaryotic-like cytoplasmic poly(A) polymerase essential in human embryonic stem cells. *Nucleic Acids Res* **48**, 2733-2748 (2020).
51. Bilska, A. *et al.* Immunoglobulin expression and the humoral immune response is regulated by the non-canonical poly(A) polymerase TENT5C. *Nat Commun* **11**, 2032 (2020).
52. Kazazian, K. *et al.* FAM46C/TENT5C functions as a tumor suppressor through inhibition of Plk4 activity. *Commun Biol* **3**, 448 (2020).
53. Grunwald, B., Schoeps, B. & Kruger, A. Recognizing the Molecular Multifunctionality and Interactome of TIMP-1. *Trends Cell Biol* **29**, 6-19 (2019).
54. Park, J. *et al.* 4-O-Carboxymethylascochlorin Inhibits Expression Levels of on Inflammation-Related Cytokines and Matrix Metalloproteinase-9 Through NF-kappaB/MAPK/TLR4 Signaling Pathway in LPS-Activated RAW264.7 Cells. *Front Pharmacol* **10**, 304 (2019).
55. Yang, C.C. *et al.* Lipopolysaccharide-Induced Matrix Metalloproteinase-9 Expression Associated with Cell Migration in Rat Brain Astrocytes. *Int J Mol Sci* **21**(2019).
56. Park, H.H. *et al.* Acetylated K676 TGFBIp as a severity diagnostic blood biomarker for SARS-CoV-2 pneumonia. *Sci Adv* **6**(2020).
57. Sun, Y. *et al.* Estrogen weakens muscle endurance via estrogen receptor-p38 MAPK-mediated orosomucoid (ORM) suppression. *Exp Mol Med* **50**, e463 (2018).
58. McPherson, R.A.P., M.R. *Henry's Clinical Diagnosis and Management by Laboratory Methods, 24th Edition*, (Elsevier Health Sciences, 2022).
59. Ruiz, M. Into the Labyrinth of the Lipocalin alpha1-Acid Glycoprotein. *Front Physiol* **12**, 686251 (2021).
60. Ligresti, G., Aplin, A.C., Dunn, B.E., Morishita, A. & Nicosia, R.F. The acute phase reactant orosomucoid-1 is a bimodal regulator of angiogenesis with time- and context-dependent inhibitory and stimulatory properties. *PLoS One* **7**, e41387 (2012).
61. Tumbarello, D.A., Temple, J. & Brenton, J.D. ss3 integrin modulates transforming growth factor beta induced (TGFBI) function and paclitaxel response in ovarian cancer cells. *Mol Cancer* **11**, 36 (2012).
62. Choi, S.I. *et al.* Involvement of TGF-beta receptor- and integrin-mediated signaling pathways in the pathogenesis of granular corneal dystrophy II. *Invest Ophthalmol Vis Sci* **51**, 1832-47 (2010).
63. Thapa, N., Lee, B.H. & Kim, I.S. TGFBIp/betaig-h3 protein: a versatile matrix molecule induced by TGF-beta. *Int J Biochem Cell Biol* **39**, 2183-94 (2007).
64. Boncela, J., Papiewska, I., Fijalkowska, I., Walkowiak, B. & Cierniewski, C.S. Acute phase protein alpha 1-acid glycoprotein interacts with plasminogen activator inhibitor type 1 and stabilizes its inhibitory activity. *J Biol Chem* **276**, 35305-11 (2001).
65. Brandow, A.M. & Liem, R.I. Advances in the diagnosis and treatment of sickle cell disease. *J Hematol Oncol* **15**, 20 (2022).
66. Nath, K.A. & Hebbel, R.P. Sickle cell disease: renal manifestations and mechanisms. *Nat Rev Nephrol* **11**, 161-71 (2015).
67. Hebbel, R.P. Beyond hemoglobin polymerization: the red blood cell membrane and sickle disease pathophysiology. *Blood* **77**, 214-37 (1991).
68. Gardner, K. & Thein, S.L. Genetic Factors Modifying Sickle Cell Disease Severity. in *Sickle Cell Anemia: From Basic Science to Clinical Practice* (eds. Costa, F.F. & Conran, N.) 371-397 (Springer International Publishing, Cham, 2016).
69. Rees, D.C., Brousse, V.A.M. & Brewin, J.N. Determinants of severity in sickle cell disease. *Blood Rev* **56**, 100983 (2022).
70. Conran, N. & Belcher, J.D. Inflammation in sickle cell disease. *Clin Hemorheol Microcirc* **68**, 263-299 (2018).
71. Kong, D.H., Kim, Y.K., Kim, M.R., Jang, J.H. & Lee, S. Emerging Roles of Vascular Cell Adhesion Molecule-1 (VCAM-1) in Immunological Disorders and Cancer. *Int J Mol Sci* **19**(2018).

72. Dworkis, D.A. *et al.* Severe sickle cell anemia is associated with increased plasma levels of TNF-R1 and VCAM-1. *Am J Hematol* **86**, 220-3 (2011).
73. Arienti, S., Barth, N.D., Dorward, D.A., Rossi, A.G. & Dransfield, I. Regulation of Apoptotic Cell Clearance During Resolution of Inflammation. *Front Pharmacol* **10**, 891 (2019).
74. Axelrod, H. & Pienta, K.J. Axl as a mediator of cellular growth and survival. *Oncotarget* **5**, 8818-52 (2014).
75. Antony, J. & Huang, R.Y. AXL-Driven EMT State as a Targetable Conduit in Cancer. *Cancer Res* **77**, 3725-3732 (2017).
76. Lee, Y.S. *et al.* Adipocytokine orosomucoid integrates inflammatory and metabolic signals to preserve energy homeostasis by resolving immoderate inflammation. *J Biol Chem* **285**, 22174-85 (2010).
77. Eriksson, O., Mohlin, C., Nilsson, B. & Ekdahl, K.N. The Human Platelet as an Innate Immune Cell: Interactions Between Activated Platelets and the Complement System. *Front Immunol* **10**, 1590 (2019).
78. Sanjabi, S., Zenewicz, L.A., Kamanaka, M. & Flavell, R.A. Anti-inflammatory and pro-inflammatory roles of TGF-beta, IL-10, and IL-22 in immunity and autoimmunity. *Curr Opin Pharmacol* **9**, 447-53 (2009).
79. Akhurst, R.J. & Hata, A. Targeting the TGFbeta signalling pathway in disease. *Nat Rev Drug Discov* **11**, 790-811 (2012).
80. Jackson, D.G. Hyaluronan in the lymphatics: The key role of the hyaluronan receptor LYVE-1 in leucocyte trafficking. *Matrix Biol* **78-79**, 219-235 (2019).
